# Fungal co-expression network analyses identify pathogen gene modules associated with host insect invasion

**DOI:** 10.1128/spectrum.01809-23

**Published:** 2023-09-01

**Authors:** Shuaishuai Huang, Xin Zhao, Zhibing Luo, Xiaohan Tang, Yonghong Zhou, Nemat Keyhani, Yongjun Zhang

**Affiliations:** 1 Key Laboratory of Agricultural Biosafety and Green Production of Upper Yangtze River (Ministry of Education), College of Plant Protection, Southwest University, Chongqing, China; 2 Key Laboratory of Biodiversity and Environment on the Qinghai-Tibet Plateau (Ministry of Education), School of Ecology and Environment, Tibet University, Tibet, China; 3 Department of Biological Sciences, University of Illinois, Chicago, Illinois, USA; Chinese Academy of Sciences, Shanghai, China

**Keywords:** transcriptome, co-expression, infection-associated modules, entomopathogenic fungi, *Beauveria bassiana*, host-pathogen interaction

## Abstract

The broad host range fungal insect pathogen, *Beauveria bassiana*, has been commercialized as an alternative to chemical insecticides for pest control worldwide. *B. bassiana* represents a unique model system with which to examine host-pathogen interactions, and a wide range of genes and processes have been studied. However, significant aspects of virulence, particularly on the genomic scale, remain poorly studied. Here, we have combined available transcriptomes with three newly generated data sets for a combined total analysis of 76 deep-sequenced samples covering growth, development, stress responses, and infection during the life cycle of *B. bassiana*. Co-expression network analyses resulted in the identification of gene modules enriched during two critical stages of the infection process, namely (i) cuticle penetration and (ii) *in vivo* hyphal body (dimorphic transition) growth capable of avoiding innate and humoral immune defenses. These analyses identify unique signatures of metabolism, signaling, secondary metabolite production, host defense suppression, membrane reorganization, effector production, and secretion for each stage, including genetic regulators and epigenetic patterns. These data provide a comprehensive framework for understanding and probing fungal adaptations to its pathogenic life cycle and expand the candidate repertoire for continued dissection of the host-pathogen interaction.

**IMPORTANCE:**

Insect fungal pathogens have evolved unique strategies for overcoming host structural and immunological defenses that span from the sclerotized cuticle to innate and humoral cellular responses. Two critical stages of the infection process involve (i) cuticle penetration and (ii) immune evasion within the insect hemocoel. A set of 76 global transcriptomic data for *B. bassiana* that include the cuticle penetration and hemocoel growth stages were analyzed for patterns (gene modules) of expression, yielding unique insights into these different life stages. These analyses integrate gene networks involved in fungal development, stress response and pathogenesis to further the systematic understanding of the global processes integral to the unique adaptation employed by fungal pathogens of insects.

## INTRODUCTION

Entomopathogenic fungi are widely distributed in diverse ecosystems, playing important roles in the regulation and turnover of insect populations worldwide (1, 2). Several entomopathogenic fungi have been successfully commercialized for the biological control of important agricultural pests, with a recent example of the long-term application of these agents for the control of rice pests showing sustainable and environmentally friendly outcomes (3). These fungi, particularly from the *Metarhizium* and *Beauveria* genera, are considered more ecologically friendly and sustainable as compared to traditional pesticides (4, 5), and thus have significant potential for increasing agricultural productivity and global food security. Significant aspects of the mechanisms by which these fungi parasitize their hosts have been characterized (6). Infection proceeds via attachment of fungal conidiospores to the insect surface, followed by germination, cuticle penetration of the integument reaching the hemocoel, proliferation within the hemocoel and invasion of surrounding tissues, and finally growth outward and sporulation on the insect cadaver (7, 8). Similar to many plant and human pathogenic fungi, entomopathogenic fungi undergo a dimorphic transition during growth in the hemocoel, producing free-floating cells termed hyphal bodies that are capable of evading host immune responses (9). In addition, as for many host-pathogen systems, entomopathogenic fungi and their insect hosts have been shown to engage in an “arms race” where the pathogen overcomes host defenses that are then under selection for improved defense, which in turn is countered by the pathogen (10). Thus far, mechanisms by which the fungus utilizes host nutrients (11, 12), modulates host immunity and defense (13), induces toxicity (14, 15), and responds to stress (16), among many others, have been characterized. Most such studies have involved gene-by-gene functional characterization, with more global overview models lacking. Thus, there is a need for greater synthesis to help classify genetic networks and traits in terms of their interconnections and/or identify unified modules, which would further our understanding of the fungal infection process and could potentially be exploited to increase the efficacy of these fungi for pest biological control.

Large-scale comparative transcriptomic studies have the potential to shed unprecedented insights into the global gene networks involved in biological processes. Currently, there are over 1,000 transcriptomic data sets of various entomopathogenic fungi available online in the NCBI SRA database. However, many of these data examine comparisons between single-gene mutants and the wild type, often under very standard *in vitro* growth conditions, i.e., in mycological media. Although lacking comprehensive integration, some of this data has been used to identify limited associations of genes or pathways to specific physiological traits (17, 18). Since gene expression changes include significant variation as well as transcriptomic shifts of the fungal cells to different physiological conditions, most transcriptomic analyses fail to examine global patterns across different fungal life stages, particularly within the context of the pathogenic process.

In this study, we generated a transcriptional co-expression network in *Beauveria bassiana* across comprehensive and systematic growth and life-stage conditions, including (i) during growth in various artificial media (nine different conditions), (ii) during the cuticle attachment and infection process, and (iii) from the *in vivo* dimorphic transitional state of the fungus, that is, the free-floating (within the insect open circulatory system) fungal hyphal bodies that are capable of evading host innate and humoral defenses. A set of 73 different transcriptomic data sets, generated using five different *B. bassiana* strains, were obtained from existing data repositories, and these data were combined with three transcriptomics data sets generated in this work, for a total of 76 data sets that were analyzed. These data revealed that expression of a set of genes (*n* = 788) specifically responding to two critical stages of the infection processes, namely (i) cuticle penetration and (ii) hemolymph colonization via fungal hyphal body development and growth. Of the identified gene data set, 269/788 (34.1%) showed tight coherence to the cuticle penetration process, and 519/788 (65.9%) showed enrichment during hemolymph colonization as compared to the 64 saprophytic growth conditions examined. These data were also categorized into the two infection-associated modules (i.e., cuticle penetration and hyphal body) when globally comparing (all saprophytic growth) aerial hyphae or liquid hyphae collected from eight artificial media. During fungal cuticle penetration, a core set of genetic/metabolic processes including amino acid degradation, amino acid/ion transportation, and the secretory system predominated, whereas in hyphal bodies, RNA processing, ion transportation, and mitochondrial metabolism processes predominated. In addition, a set of 10 genes highly enriched in hyphal bodies was identified. Our analyses help dissect the genetic determinants that affect fungal fitness for varied physiological traits, focusing on the cuticle penetration and hemolymph colonization stages that are unique to fungal pathogens of insects. These analyses provide a framework for organizing genetic networks and processes for successful fungal infection of insect hosts and identify additional candidate genes implicated in these processes.

## RESULTS

### A pipeline for the identification of pathogenicity-associated gene modules for *B. bassiana*

**Fig 1:**
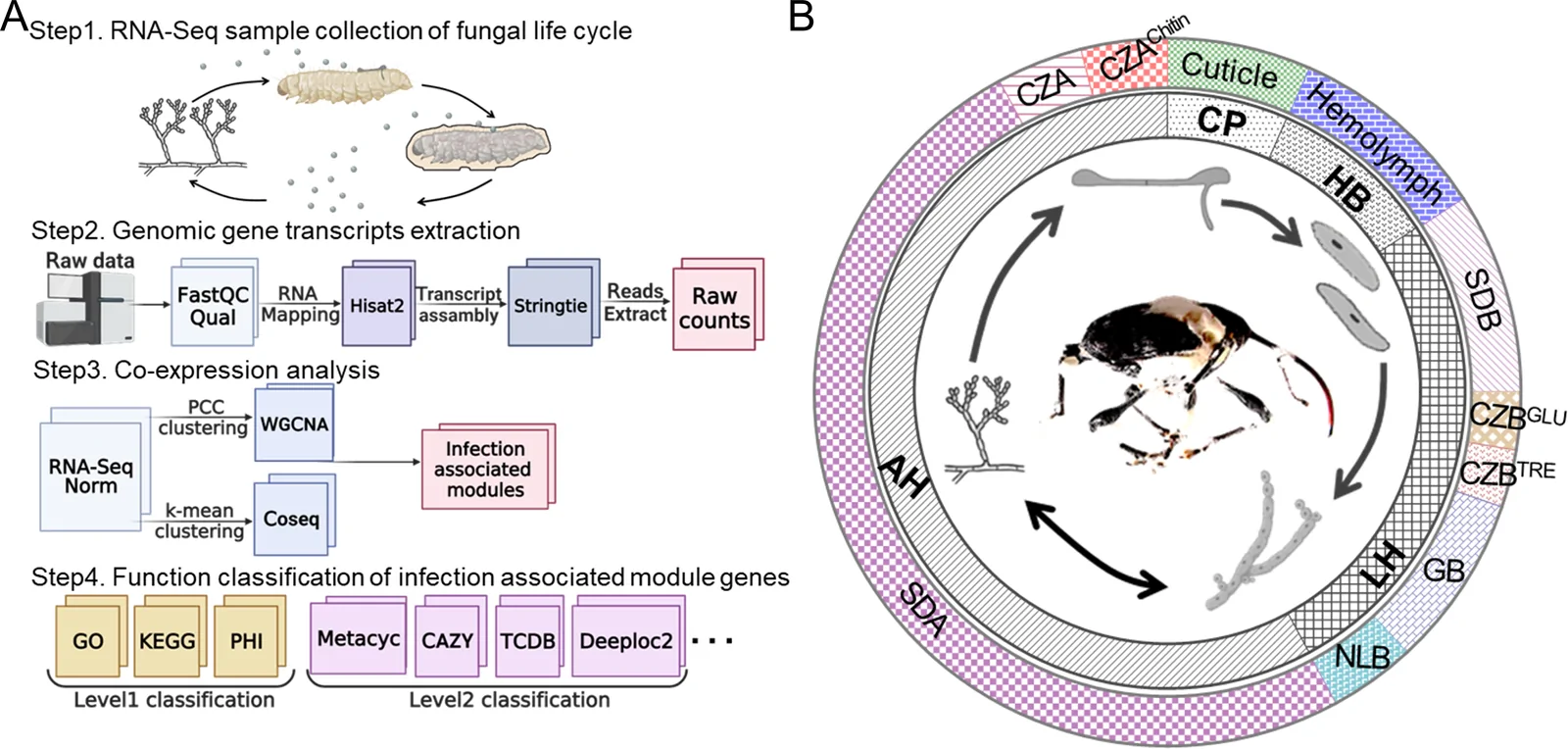
Working pipeline and sample collections for identification of infection-associated gene modules. RNA-seq samples derived from different cell types in the *B. bassiana* life cycle. (**A**) Overview of bioinformatic analysis workflow. (**B**) Physiological niches of collected RNA-seq samples. Fungal cell types were annotated as: AH, aerial hyphae; CP, cuticle-penetrated cells; HB, hyphal bodies; and LH, Liquid hyphae. Nutrition bases include SDA, SDB, GB (germination broth), CZA, NLB and chitin, trehalose, glucose-supplemented CZA or CZB medium for CZA-chitin, CZB_TRE, and CZB_GLU, respectively. The proportion of each sample type was plotted accordingly.

To test and probe specific trait-associated gene modules for *B. bassiana*, a four-step transcriptome-mediated co-expression analysis pipeline was generated (Fig. 1A). This included (i) data collection, (ii) transcript extraction, (iii) co-expression analysis, and (iv) functional characterization of module genes. A total of 76 transcriptomic (RNA-seq) data sets were retained that covered both public-released *B. bassiana* RNA-Seq transcriptomes (73, November 2022, National Center for Biotechnology Information, NCBI) as well as three data sets generated in this study (Fig. 1B; Table S1). The combined data set was derived from five different *B. bassiana* strains and included a total of 10 different conditions or cell types as follows: (i) *B. bassiana* strain ARSEF 2860 grown in liquid media: (a) Sabouraud dextrose broth (SDB), (b) germination broth (GB), (c) nitrogen-limited broth (NLB), (d) Czapek-Dox broth + glucose (CZB + GLU), (e) CZB +trehalose (CZB + TRE), mycelia derived from agar plates as follows: (f) SDA, (g) CZA + chitin, (h) CZA, and (i) hyphal bodies isolated from the hemolymph of infected *G. mellonella* (HB 1–4, 6–7); (ii) *B. bassiana* strains GXsk1011, CQsk1209, and GXtr1009 grown under conditions of cuticle penetration, that is, grown in the presence of insect cuticles derived from silkworm (CP1-3), *Helicoverpa armigera* (CP-4), and hawk moth (CP-5), respectively; (iii) *B. bassiana* strain CGMCC 7.34 grown in (a) SDB for 3 d, (b) hyphal bodies isolated from the hemolymph of *G. mellonella* 2 d after infection (HB-5), and (c) 1/4 SDY broth for 3 d. Data sets corresponding to (i) and (ii) were obtained from the NCBI database as indicated, and the data sets corresponding to (iii) were generated as part of this study. In total, five data sets corresponded to the cuticle penetration stage, seven data sets to hyphal bodies, 20 to hyphal growth in liquid media, and 44 to hyphal growth on solid substrates.

**Fig 2:**
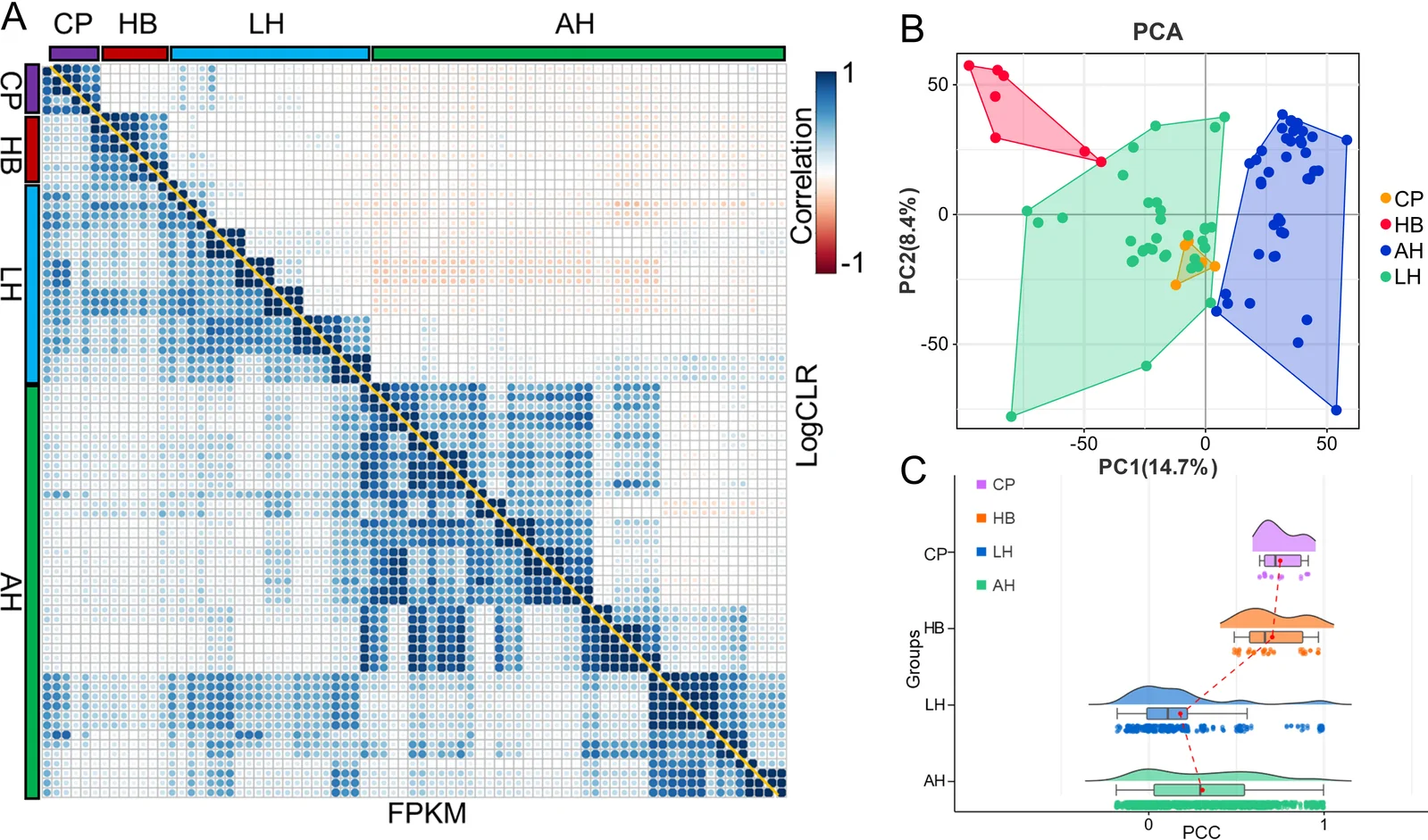
Correlations of RNA-Seq samples within or between growth conditions. (**A**) Correlation matrix of normalized RNA samples for FPKM (down-left) and LogCLR (up-right), respectively. The bars on the left and top of the matrix are labeled according to growth condition: CP, cuticle penetrations; HB, hyphal bodies; LH, liquid hyphae; AH, aerial hyphae. Sample correlation intensity was colored as bars. (**B**) Principal component analysis (PCA) of RNA-seq samples. Transcriptomes from different groups were decorated and enclosed in different colors as indicated. (**C**) Distribution of PCCs of RNA samples inside given groups. The mean PCC value was connected as a red dashed line, and sample groups were decorated accordingly.

A uniform pipeline (as detailed in the Materials and Methods section) was used to assess the global expression (in reads) of all 10,365 genes in *B. bassiana* genome across the 76 sample data sets used. Gene expression with zero reads across more than five samples was discarded from further analysis. For robust co-expression analyses, five different normalization algorithms were tested, including those without data centralization processing. These algorithms included (i) fragments per kilobase of transcript per million mapped reads (FPKM) (19), (ii) transcripts per kilobase million (TPM), (iii) Up-quantile normalized reads (UQ), (iv) log *trans* FPKM + TMM norm + median polish (FPKM^t1^), and (v) Log *trans* raw reads + TMM norm + median polish (LogCLR) (20). A PCC hierarchical clustering heatmap was generated to visualize sample similarity (Fig. 2A; Fig. S1). These analyses revealed that the LogCLR normalization outperformed the other algorithms, with more significant correlations seen after the LogCLR normalization as compared to the others, especially the PCC matrix of FPKM. Hence, LogCLR was deployed for further analyses of co-expression networks. Principal component analyses (PCA) plots confirmed that data set conditions clustered into three distinct groupings (Fig. 2B). These included one group consisting of the hyphal body data sets (red in Fig. 2B diagram) and then a clear separation between liquid-grown hyphae/mycelia (LH, green) and aerial hyphae/conidia isolated from agar media conditions (AH, blue). Intriguingly, a small nexus of points corresponding to cuticle penetration cells (CP, yellow) clustered in a region within the liquid hyphae area. To explore RNA-seq sample similarity inside given conditions, Pearson’s correlation coefficient (PCC) analyses were performed and indicated high PCC values (i.e., strong correspondence of gene expression between the samples compared) for the CP (0.75 ± 0.1) and HB (0.70 ± 0.16) groups (Fig. 2C). However, for the liquid hyphae (LH) and aerial hyphal (AH) samples, PCC values were much lower at 0.18 ± 0.29 and 0.31 ± 0.3, likely reflecting the heterogenicity of the samples since they were derived from a variety of different growth conditions. Samples from different *B. bassiana* strains (e.g., 2860, GXtr series, CGMCC 7.34) collected in liquid hyphae or CP conditions showed an insignificant scattered pattern in the PCA plot (Fig. 1B) and were positioned closely to one another as compared to samples from HB or AH condition, indicating a minor interference of strain variations in our analysis.

**Fig 3:**
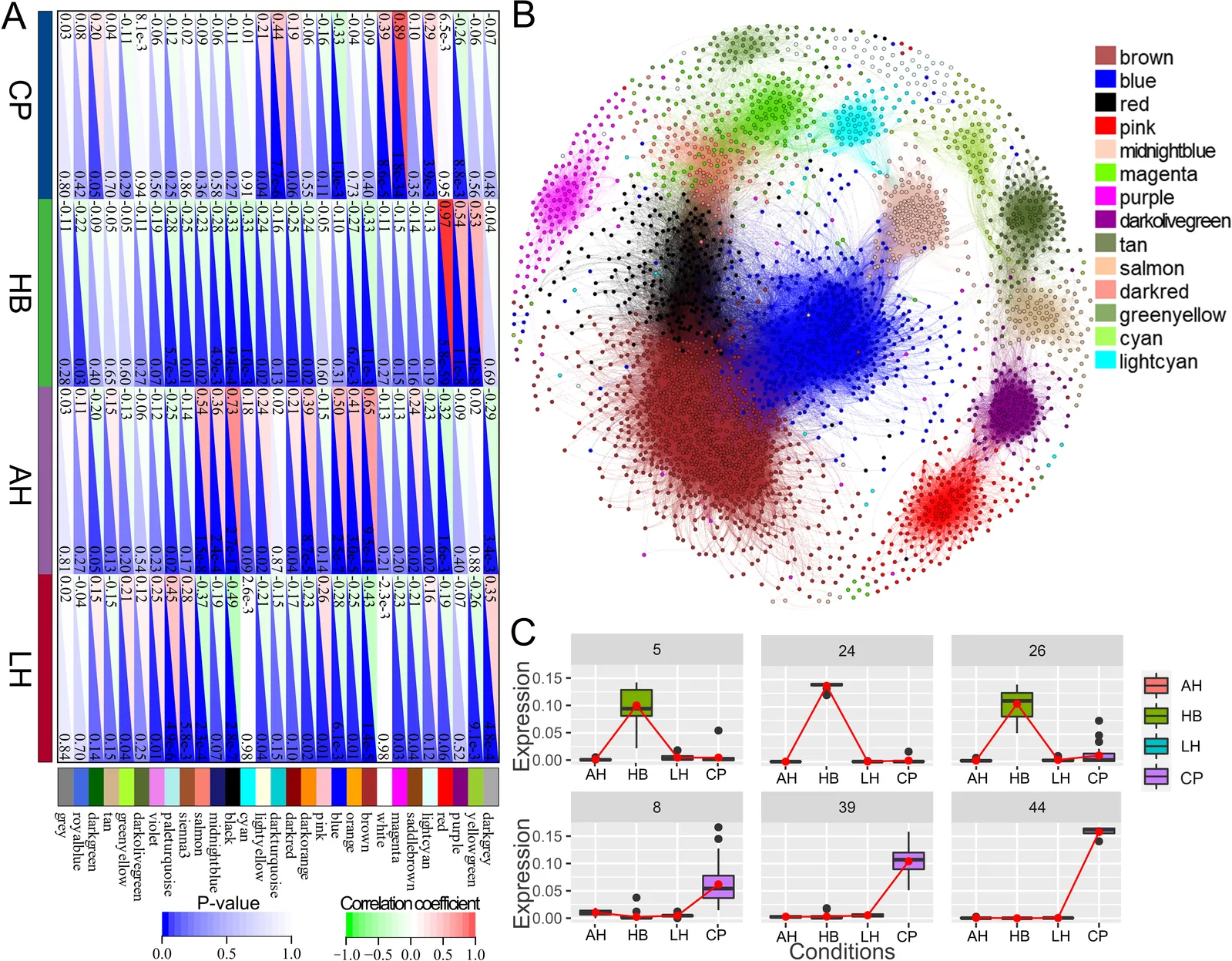
Discovery and illustration of infection-associated modules. (**A**) Correlation matrix of WGCNA generated co-expression modules with physiological traits. Correlation coefficients of PCC were listed and decorated red to green for positive and negative correlations in the up-left triangle in each correlation box, and correlation *P*-value was marked and decorated accordingly in the down-right panel. (**B**) *B. bassiana* GGM gene co-expression network. Only modules with 100+ genes were shown due to the limited space. Different modules were color-coded as indicated in the legend. Nodes refer genes, co-expressed genes were interconnected with line color similar to each modules; the size of node represents connection counts. Genes with PCC threshold of 0.6 were used for presentation. (**C**) Expression profile of infection-associated module genes detected by Coseq, mean expression value of module genes in the given condition was linked with solid red. Cell types were color-coded as indicated. The number on top refers to the module rank number in the analysis.

**Fig 4:**
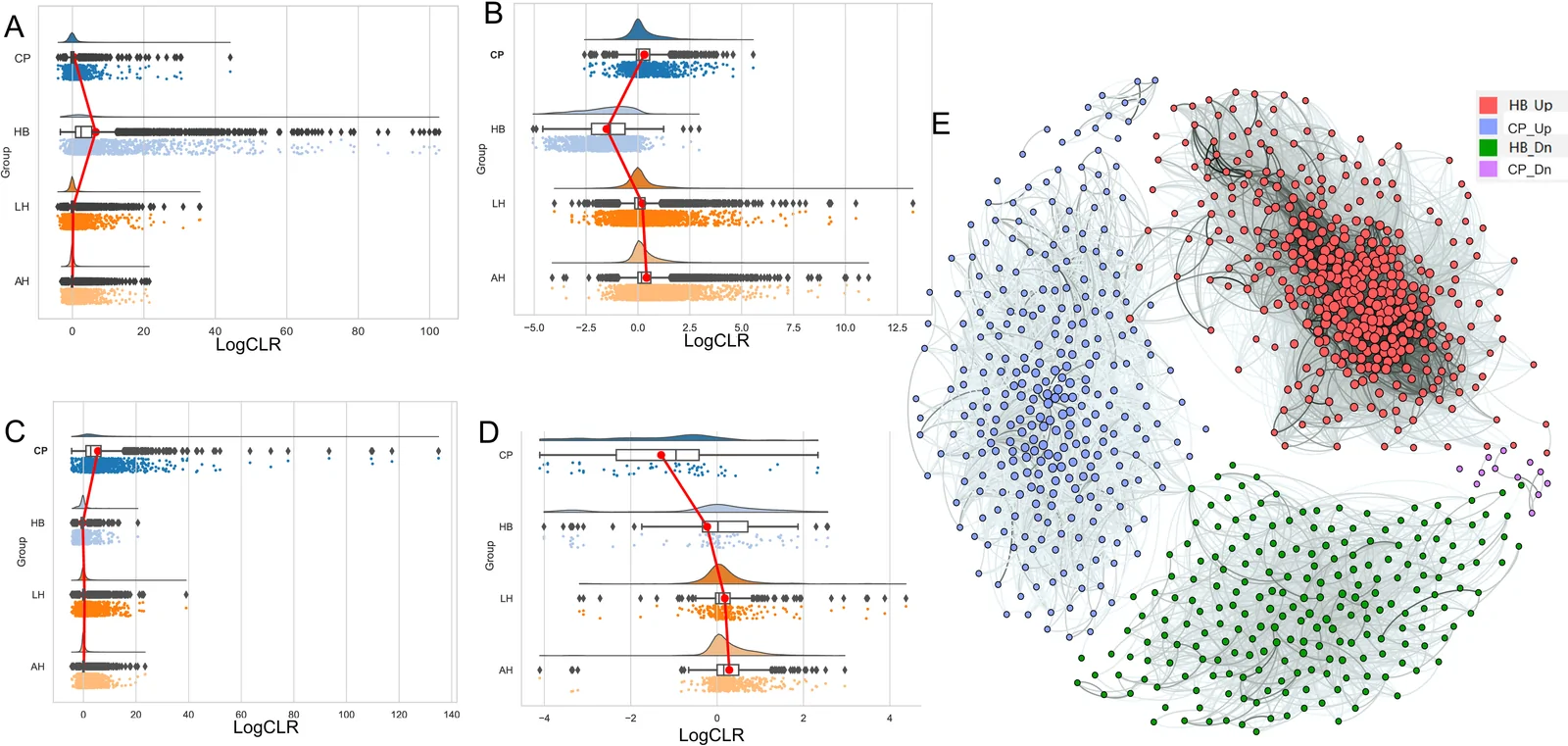
Global expression and co-expression networks of infection-associated modules. Two infection modules were separated into four subgroups: HB-Up and HB-Dn refer to the subgroup module genes that are mainly expressed or depressed in hyphal bodies, respectively; CP-Up and CP-Dn represent CP-associated module subgroups that were overexpressed or silent in CP trait. Expression profile of genes in subgroups HB-Up (**A**), HB-Dn (**B**), CP-Up (**C**), and CP-Dn (**D**) in four conditions were plotted and color-coded accordingly. Mean expression value of module genes in each condition was linked in solid red. (**E**) Co-expression network of four module subgroup genes associated with infection. Positive gene-gene PCC correlations of over 0.7 were used for illustration, size of node represents connection counts, and connections between genes indicate a co-expression relationship.

**Fig 5:**
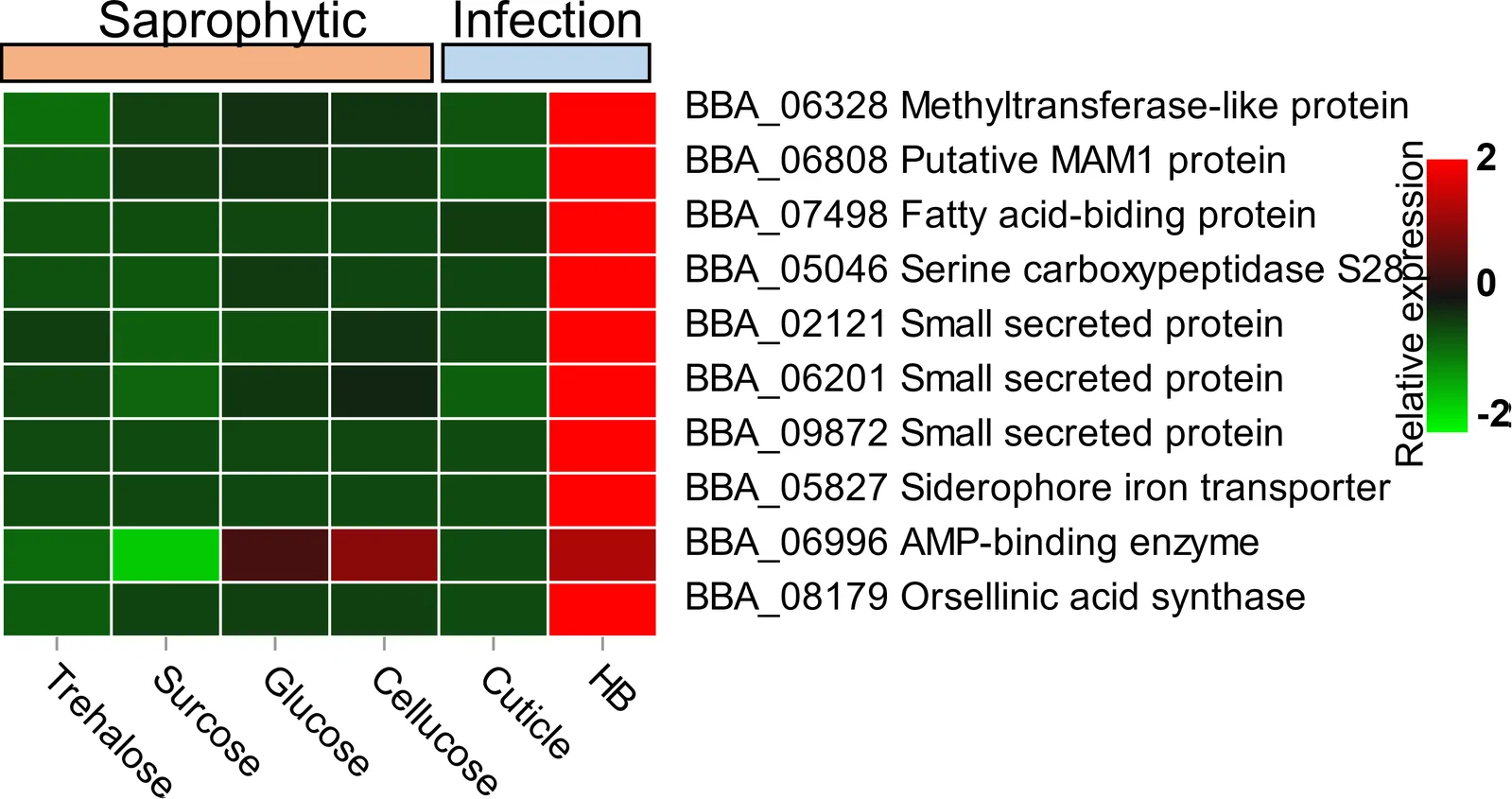
The RT-qPCR validation of the expression profile of 10 genes that are mainly expressed in HB condition. Samples were divided into saprophytic and infection groups and marked as bars in different colors. Trehalose, sucrose, glucose, and cellulose refer to CZB broth supplemented with carbohydrates previously listed as a solo carbon source [3% (vol/wt)]. Cuticle indicates fungal cells incubated with silkworm cuticle; HB refers to hyphal bodies collected from *B. bassiana*-infected *G. mellonella*. Relative expression refers to *z*-score normalized expression between samples.

Co-expression modules (sets of genes) were detected using mixture models with two programs: WGCNA (21) and Coseq (Gaussian mixture implemented) (22) that utilize PCC hierarchical clustering (PCC-based) and K-mean-based clustering algorithms, respectively. WGCNA analyses revealed 29 co-expression gene modules (color coded in Fig. 3A), with one module (“magenta”) tightly correlated to CP samples (0.89, 202 genes). Hyphal body (HB) samples harbored three correlated modules with threshold PCC ≥0.5, module “red” (0.97, 218 genes), “purple” (0.54,167 genes), and “yellow-green” (0.53, 32 genes). No modules were detected as expressed in both HB and CP conditions with the given threshold of PCC ≥0.5. Four modules (brown, 1,029 genes; blue, 575 genes; black, 306 genes; and salmon, 121 genes) showed PCC ≥0.5 in the aerial hyphal data sets, and no module reached PCC ≥0.5 for the liquid hyphal samples. Co-expression network groupings of the WGCNA analyses (Fig. 3B) were also generated, and modules with over 100 genes were used for visualization. HB-associated modules of red, purple, and yellow-green were tightly interconnected, and module magenta was scattered and floated aside others, indicating a discrete global gene expression profile. Four modules with high affinity to condition AH (Brown, Black, Blue, and Salmon) are the most abundant modules and closely clustered with each other. A K-mean clustering of co-expression clusters with Coseq revealed three clusters (No. 5, 24, 26) that covered 421 genes that were highly overexpressed in HB samples, and the other three clusters (No. 8, 39, 44) accounting for 193 genes with elevated transcripts abundance (transformed to LogCLR) in CP samples (Fig. 3C). WGCNA and Coseq detected CP- and HB-associated modules exhibited moderate consistency, with 55.4% overlap in CP-associated modules and 48.0% overlap in the HB modules, indicating good confidence in trait-associated module discovery by two clustering algorithms. CP and HB modules were then first screened with a PCC threshold of ≥0.6, and subsequently manually curated to capture 289 transcripts specifically or largely (over)expressed in the CP samples (CP-Up) and 12 transcripts specifically downregulated (CP-Dn). For HB samples, 366 transcripts were identified in the specific/high expression category (HB-Up), and 213 transcripts that were downregulated (HB-Dn). General module gene annotation and classification of these four groups are given (Fig. S2; Table S3 to S6). The expression profiles of all genes in subgroup HB-Up were plotted under each condition (CP, HB, LH, and AH), showing a large increase in expression in the HB condition (average LogCLR = 2.7 ± 1.1) as compared to the other three conditions (CP 1.6 ± 0.65, LH 1.57 ± 0.52, 1.55 ± 0.39) (Fig. 4A). These data show the collective expression (in terms of LogCLR on the x-axis) of all HB-Up genes with each point referring to the expression values of select genes in the HB-Up subgroup. Line connections indicate mean value connections, that is, the variation in expression between different groups. The distribution curves on top of each panel indicate the average data distribution in the dimension of expression. Similarly, the HB-Dn data showed expression module genes that had relatively decreased expression levels (−1.51 ± 1.16) in HB as compared to the CP data (0.31 ± 0.86), LH (0.21 ± 0.0.99), and AH (0.42 ± 0.84) (Fig. 4B). In addition, the expression of genes in subgroup CP-Up was more abundant in the CP data set (5.56 ± 9.7) as compared to the HB (−0.26 ± 1.69), LH (0.43 ± 1.99), and AH (0.18 ± 1.20) (Fig. 4C). The CP-Dn-enriched gene data set exhibited weak expression in condition CP (−1.30 ± 1.51) as compared to HB (−0.23 ± 1.58), LH (0.18 ± 0.79), and AH (0.28 ± 0.59) data sets (Fig. 4D). All four subset module genes exhibited significant differential expression profiles compared to the other conditions (data sets) (*P <* 0.001) (*t*-test), which was visualized in a co-expression network plot of these four groups showing that transcripts belonging to specific modules were clustered (Fig. 4E). All four modules were scattered from each other, and module genes in HB-Up were more interconnected as compared to the other three subgroups. To validate the modules, the expression patterns of 10 randomly selected transcripts from the HB-UP module were examined by qRT-PCR (Fig. 5). Consistently, from the co-expression analyses, the expression of all 10 candidates was generally low in all conditions except in hyphal bodies, with a slight upregulation of the AMP-binding protein (BBA_08179) under cellulose and glucose growth conditions.

### Analyses of the co-expression module genes

**Fig 6:**
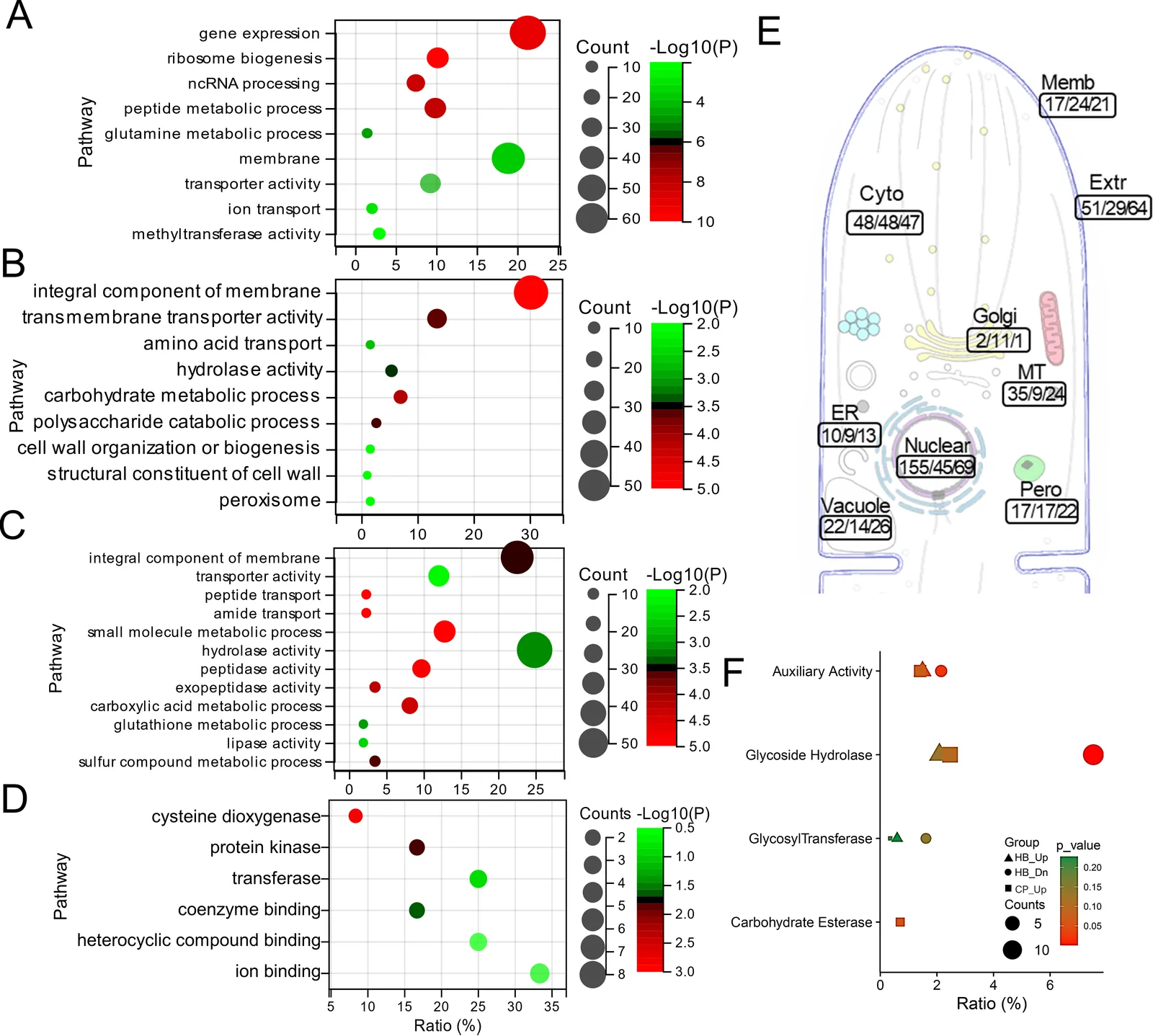
Enrichment analysis of infection-associated module genes. Four infection-related subgroups were identified and categorized as detailed in the experimental protocols and results section. GO analysis of module genes in subsets of HB-Up (**A**), HB-Dn (**B**), CP-Up (**C**), CP-Dn (**D**) was shown, and enriched GO catalogs were selected for presentation. The size of the symbols in the plots reflects the relative number of module genes in the indicated category. Confidence intervals (*P*-values) of assignments are coded in color from green to red as indicated. (**E**) Subcellular localization of three infection-associated module genes (HB-Up, HB-Dn, and CP-Up). The number of module genes that fell into eight cellular compartments predicted by deeploc2 was calculated and illustrated. Cyto, cytoplasm; Memb, membrane; Extr, extracellular; ER, endoplasmic reticulum; Pero, peroxisome; Golgi, golgi apparatus; Nuclear, cell nucleus. (**F**) Enrichment of module genes in Carbohydrate-Active enZYmes (CZAY) Database. The size of symbols indicates enriched gene number, groups were decorated in three shapes: triangle (HB-Up), circle (HB-Dn), and square (CP-Up). Confidence intervals (*P*-values) were synergistic to color bar from green to red, as indicated.

To explore gene networks implicated in the CP and HB modules, general gene annotation analyses (GO, KOG, and PHI) were performed (Fig. 6; Fig. S3). In GO analyses, the HB-Up module was found to be highly enriched in gene expression and translation (71 genes, *P* < 0.01), including ribosome biogenesis (34 genes) and noncoding RNA (ncRNA) processes (25 genes) (Fig. 6A). Significant shifts in nutrient uptake systems were also noted, with 63 membrane proteins (31 corresponding to transporters) in the HB-Up modules and another set of 56 membrane proteins (25 transporters) enriched in the HB-Dn module. The HB-Up modules were also enriched in nitrogen metabolism (92 transcripts), which highlighted peptide biosynthesis (33 transcripts) (*P <* 0.01). For the CP-Up module, 25 peptidases, five lipases, and 58 membrane proteins including 31 transporters were enriched (*P* < 0.01, Fig. 6C), consistent with the requirement for the fungus to penetrate and degrade the insect cuticle at this stage. Cysteine dioxygenase and protein kinases were overrepresented in module CP-Dn (Fig. 6D). Analyses via KOG annotation further confirmed metabolic changes in the HB and CP module genes (Fig. S3A). These analyses revealed a comparatively high level of posttranslational modification activity (22 and 24 genes, respectively) in the CP-Up and HB-Up modules (*P* < 0.01). Pathogen-host interaction (PHI) database annotation revealed 127 transcripts in the HB-Up, 84 in the HB-Dn, 121 in the CP-Up, and 3 in the CP-Dn modules, which accounted for 38, 45, 47, and 25% of the total genes given in each module subgroup, respectively (Fig. S3B). The infection-associated modules annotated via the PHI database were marked into the categories of loss of pathogenicity, unaffected/reduced virulence, increased virulence, and unaffected virulence. Of note, HB-Up (52 genes) and CP-Up (52 genes) active module genes were highly enriched in terms of reduced virulence, while HB-Dn contained 29 genes in this category, and none were detected in module CP-Dn. However, only the HB-Up module contained transcripts belonging to the lethal (10 genes) and effector (two genes) categories. Subcellular localization mapping of infection-associated module genes was also performed (Fig. 6E). For the HB-Up, 155 genes (46%) showed nuclear localization and 35 mitochondrial, with CP-Up showing 69 nuclear and 24 mitochondrial localized gene products. HB-Dn showed 45 in the nucleus and nine in the mitochondria, whereas CP-Dn showed four in the nucleus and two in the mitochondria. The HB-Up and CP-Up modules showed high secretory activity with 51 and 64 proteins, accounting for 15.3% and 24.9%, respectively, in each given module. HB-Dn, however, was enriched in Golgi activity with 11 genes downregulated, whereas HB-Up contained two Golgi-associated protein products and CP-Up only 1.

### Metabolic processes involved in fungal pathogenicity

Two important aspects of hyphal body production are shifts in carbohydrate utilization and a remodeling of the cell wall, presumably to avoid host immune defense. Consistent with this transition, significant changes were seen in transcripts involved in cell wall-related polysaccharide metabolism as analyzed using the carbohydrate-active enzymes (CAZY) database (Fig. 6E). The HB-Dn module contained 15 glycoside hydrolases (GH) (*P* < 0.001) and three glycosyltransferases (GT), whereas HB-Up yielded 7 GH and 2 GT members, and CP-Up contained 7 GH, 1 GT, and two carbohydrate esterases (GE). The clade of auxiliary activity (23) refers to GMC oxidoreductases that facilitate polysaccharide degradation by GH, and with respect to these, five were found in HB-Up, four in HB-Dn, and four in CP-Up. Two adhesions were also seen in HB-Up, whereas three glycosyl phosphatidylinositol-anchored proteins, involved in cell surface protein modification, were found in CP-Dn.

### Alterations in carbohydrate and amino acid metabolism during host infection

**Fig 7:**
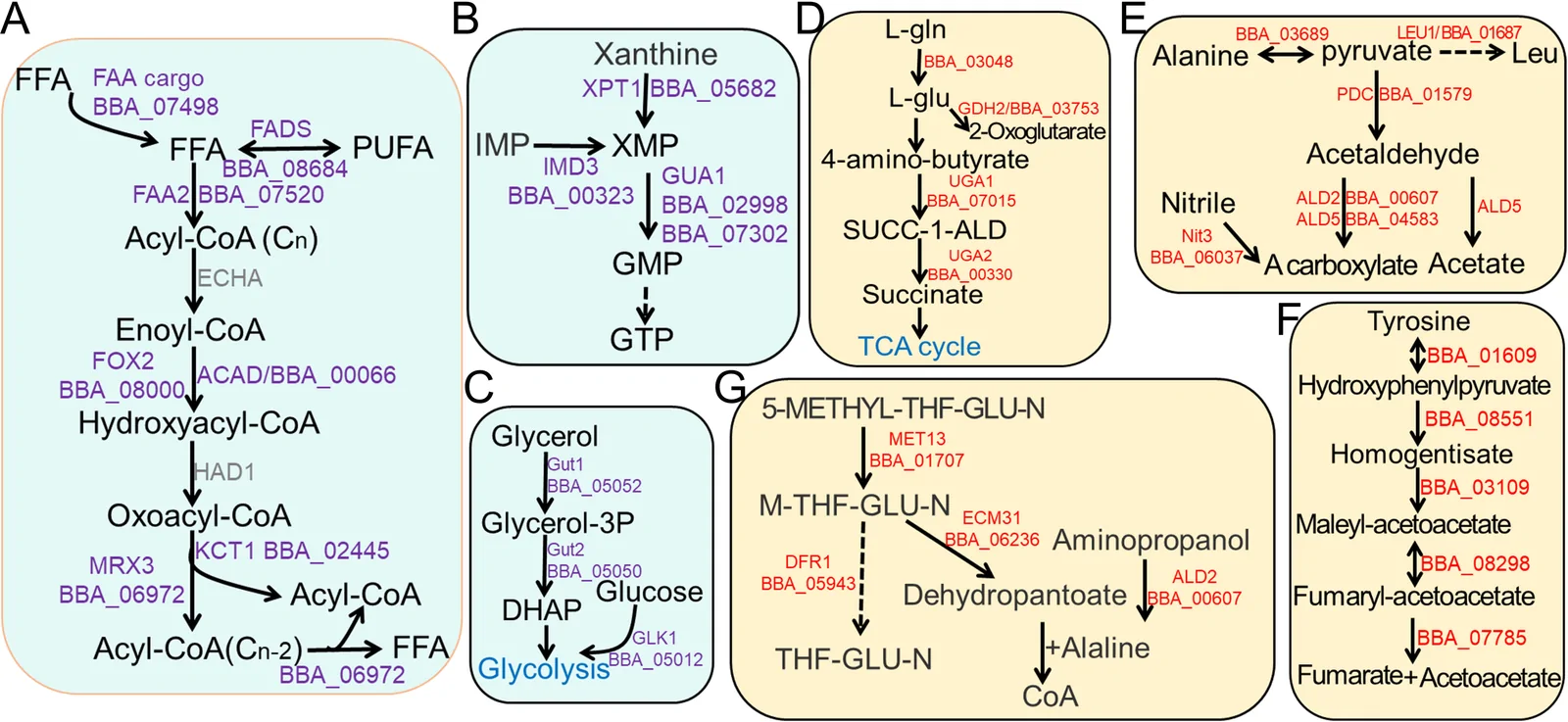
Metabolic pathway analysis of infection-associated module genes. Module genes were mapped to yeast metabolic pathways and pathways in the Metacyc database and overrepresented pathways were selected for illustration. The partially activated pathways of fatty acid degradation (**A**), GTP synthesis (**B**), and glycolysis (**C**) in HB-Up subgroup were illustrated. The active pathways in cuticle penetration (CP-Up) were shown as indicated. (**D**) Glutamic acid degradation pathway. (**E**) Pyruvate metabolic pathway. (**F**) Tyrosine degradation pathway. (**G**) Coenzyme biosynthesis pathway. The known homologs of module genes in yeast were listed as indicated or as sole *B. bassiana* genes after being mapped to Metacyc. Abbreviations of chemicals listed were annotated in Metacyc databases. Multiple reactions in between substrates were linked with a dotted arrow, while reversible reactions were shown as double arrows.

Members in glycolysis and the tricarboxylic acid (TCA) cycle were enriched in the HB-Up module, including glucokinase, glycerol kinase, glycerol-3-phosphate dehydrogenase, and fructose-1-phosphatase (Fig. 7C), and three genes in folate-mediated one-carbon metabolism (*MTD1/DFR1/purU*) were activated in the HB-Up module, while in the HB-Dn module, two glycolysis-inhibiting/competitive enzymes, pyruvate kinase and 2-deoxyglucose-6-phosphate phosphatase, were present (Tables S4 and S5). Transcripts corresponding to fumarate hydratase in the TCA cycle, hexokinase in glycolysis, 6-phosphofructo-2-kinase for regulation of glycolysis, D-galacturonic acid reductase in galacturonate catabolism, along with three enzymes in host urate degradation to glyoxylate, including two allantoate amidohydrolases (*DAL2/DAL3*) and one HIU hydrolase, were found in the CP-Up module (Table S3).

### Degradation of amino acid pathways is activated during cuticle penetration

The CP-Up module contained four transcripts involved in glutamate/glutamine catabolism related to the TCA cycle (Fig. 7D), as well as alanine/leucine to acetate (five genes) (Fig. 7E) and degradation of tyrosine (all five enzymes of the pathway) (Fig. 7F). Enzymes involved in vitamin and/or coenzyme biosynthesis, with three core genes in precursor acetyl-CoA biosynthesis, were found in the CP-Up module (Fig. 7G). In addition, the CP-Up module contained two genes involved in GABA catabolism and one in GABA regulation, genes involved in histidine/glycine conversion to thiamine (*Thi4* and *Thi13* and the *Thi2* regulator), two core enzymes involved in methionine biosynthesis (*Met6* and *Sah1*) as well as the *Met32* regulator (Table S3). In the HB-Up module, amino acid degradation/biosynthesis transcripts included glutaminase, three aminotransferases, one asparagine synthase, and one prephenate dehydratase (*PHA2*), with transcripts involved in amino acid to nucleotide conversion including four genes enriched in the generation of GTP and one carbamoyl-phosphate synthase involved in CTP synthesis (Fig. 7B).

In terms of lipid metabolism and fatty acid amine (FAA) degradation, the HB-Up module contained five genes involved in β-oxidation (*FAA2*, *FOX2*, *ACAD*, *MRX3*, *KCT1*), one FAA-binding protein, one fatty acid desaturase, and one thioesterase responsible for hydrolysis of long-chain acyl-CoA to FAA and acetyl-CoA, as well as two genes (*Erg3* and *Erg10*) in ergosterol biosynthesis (Fig. 7A). Genes involved in fungal lipid metabolism were also found in the CP-Up module, including three thiolases, two acetyl-CoA acetyltransferases, two acyl-CoA-dehydrogenases, as well as the *Erg6* sterol 24-C-methyltransferase and the *Erg3* sterol desaturase involved in ergosterol biosynthesis.

### Nutritional influx changes during host invasion

**Fig 8:**
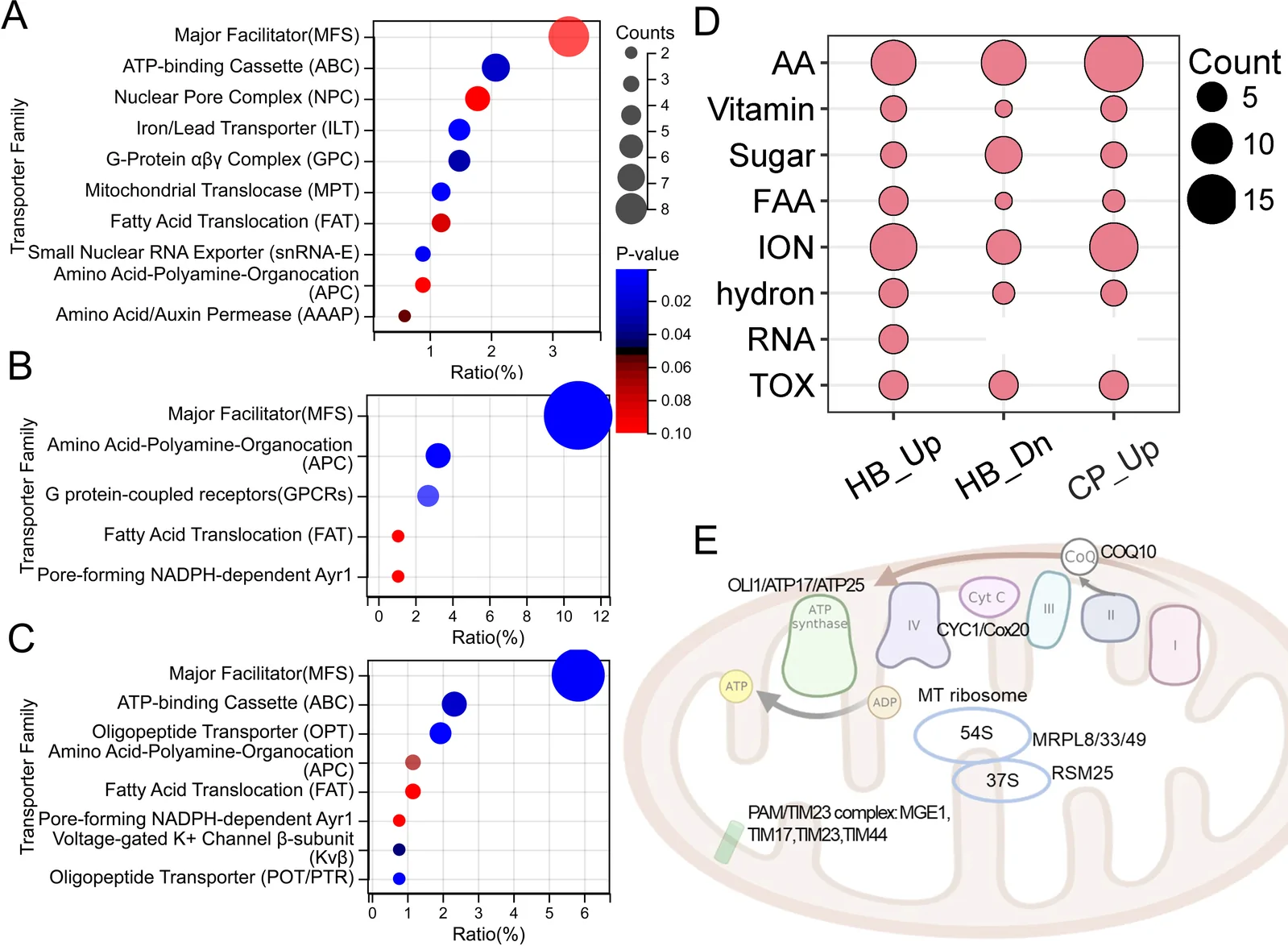
Analysis of transporters and mitochondrial function in infection-associated modules. Module genes were mapped to Transporter Classification Database (TCDB), and over-presented transporter catalogs were selected for enrichment illustration: (**A**) HB-Up, (**B**) HB-Dn, and (**C**) CP-Up. The size of nodes reflects the number of genes enriched in given categories. Confidence intervals (*P*-values) were colored from blue to red as indicated. (**D**) Enrichment of transporter substrates in three infection correlated modules. Node size refers to the number of enriched module genes. AA, amino acids; FAA, fatty acids; ION, ions; Hydron, hydrogen ion; TOX, toxins. (**E**) Active MT genes during hemolymph colonization. Yeast homologs of MT genes in the HB-Up module were used for illustration, including members in electron transfer chain, MT ribosome, and inner membrane transporters.

Membrane transporter-mediated nutrient uptake, including metal influx and toxin secretion, is critical for the success of fungal infection. The HB-Up, CP-Up, and HB-Dn infection-associated module genes were analyzed using the Transporter Classification Database (TCDB) (Fig. 8A through C). Both major facilitator superfamily (MFS) and ATP-binding cassette (ABC) transporters were highly enriched, with transcripts corresponding to 91 such transporters in the HB-UP, 61 in the HB-Dn, and 83 in the CP-Up modules (Fig. 8A through C). Ion transporters were highly enriched in HB-Up, including five iron/lead transporters (ILT), two iron hydroxamate MFS transporters, as well as separate Zn^2+^, Ca^2+^, Na^+^, and Mg^2+^ transporters. HB-Dn included one each of Fe^3+^, Zn^2+^, Cu^2+^ transporters. In the CP-Up module, two iron hydroxamate, two K^+^, two Zn^2+^, and two Ca^2+^ transporters were identified.

Amino acid uptake systems were also highly enriched; of the 50 substrate-annotated transporters found in the CP-Up module, 20 were involved in amino acid or polypeptide uptake, including aspartic acid, lysine, GABA, glutathione, and others (Fig. 8D). For the HB-Up module, a more limited number (11 total) of amino acid transporters were identified, compared to the six amino acid/peptide transporters seen in the HB-Dn module. Both the HB-Up and CP-Up modules contained three sugar transporters (different ones for each module), whereas HB-Dn contained seven MFS transporters for various mono/oligosaccharides. The HB-Up data set contained five members of the G-protein αβγ complex-coupled receptor pathway, whereas the HB-Dn module contained five different G protein-coupled receptors (GPCRs) (Fig. 8B). Of note, four transporters linked to ncRNA/mRNA were found in the HB-Up data set (Fig. 8A). In addition, HB-Up contained four FAA transporters and four toxin transporters, and CP-Up contained two and four FAA and toxin transporters, respectively.

### Mobilization of mitochondria/energy occurs during fungal colonization of host niches

Thirty-five mitochondrial genes were enriched in the HB-Up module (Fig. 8E; Table S4). These included genes involved in the electron transport chain, for example, three cytochrome P450 genes, one CoQ and three ATP synthetases, a mitochondrial complex I assembly (CIA30), and three PAM/TIM23 complex members that govern protein influx from the inner mitochondrial membrane. In addition, four mitochondrial ribosome members and three RNA synthesis/maturation genes were also enriched in the HB-Up module, as well as four chaperonins (HSP10/HSP60/SSC1/MGE1) that mediate mitochondrial protein synthesis and transport were also found. The HB-Up module also contained a transferase-mediated acetyl-CoA or FFA import gene involved in carnitine biosynthesis (succinyl-CoA:3-ketoacid-coenzyme A transferase), glutaminase, a mitochondrial intermediate peptidase, aspartate aminotransferase, and two apoptosis-inducing factors (AIFs) involved in programmed cell death. In HB-Dn, CP-Up, and Cp-Dn, only 9, 24, and 2 mitochondrial-related genes were identified, respectively. These included, for HB-Dn: two genes in SM biosynthesis (4′-phosphopantetheine transferase, short-chain dehydrogenase), a peptidase, a mitochondrial K^+^/H^+^ exchanger pump and an activating signal co-integrator, and a transcript functioning in DNA damage repair were detected; for CP-UP: five genes in amino acid metabolism, seven genes in lipid or coenzyme metabolism, and three genes in secondary metabolite biosynthesis were found; and for Cp-Dn: a pyridoxamine 5'-phosphate oxidase in PLP biosynthesis as well as a salicylate hydroxylase were enriched.

### Secondary metabolism fluctuation during infection

**Fig 9:**
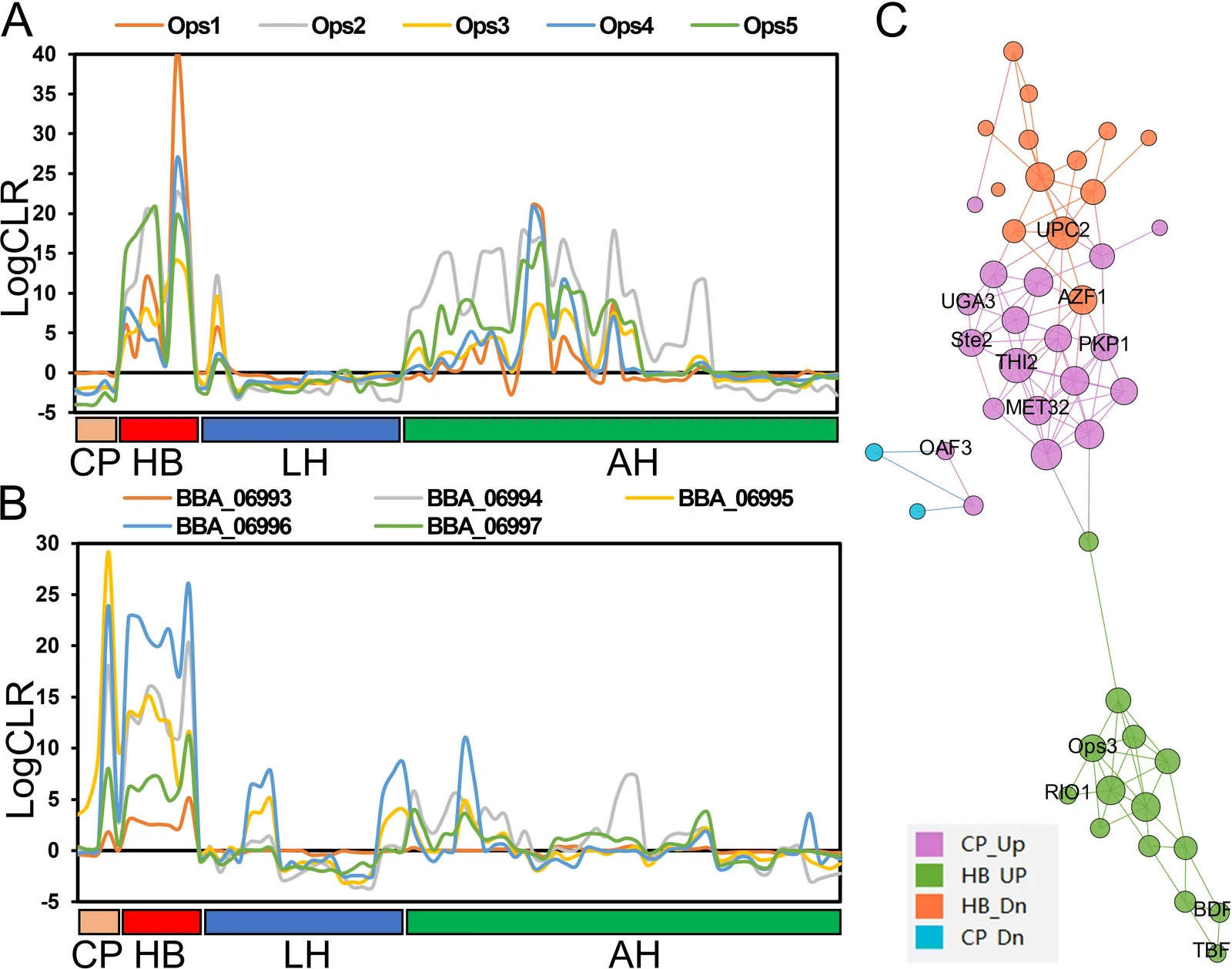
Global expression profile of two secondary metabolite biosynthesis clusters and co-expression networks of regulators in infection-associated modules. (**A**) Oosporein biosynthesis gene cluster. (**B**) A putative siderophore biosynthesis gene cluster. Normalized LogCLR values were illustrated as expression values. Samples were clustered as condition of CP, HB, LH, and AH. (**C**) Co-expression network of transcription factors and kinases in four infection-associated modules. Known/homologous genes were annotated as indicated.

In the HB-Up data set, two secondary metabolite (SM) clusters. including the polyketide synthase (PKS)-containing cluster for biosynthesis of oosporein involved in host immunity and suppression of competing microbes, and a five-gene cluster including two non-ribosomal peptide synthases (NRPS) involved in biosynthesis of the siderophore aerobactin (Fig. 9A and B). Five of the core genes in the oosporein biosynthetic cluster were tightly co-expressed in HB-Up, although some appeared randomly activated in various AH samples under specific nutrient conditions. In addition, SM-related genes found in the HB-Up module included two *O*-methyltransferases, two monooxygenases, and two cytochrome P450 enzymes. In the HB-Dn data set, two core members involved in the lovastatin siderophore biosynthetic cluster (lovastatin nonaketide synthase and an EF-hand calcium-binding domain protein), as well as one additional PKS gene, two cytochrome 450 enzymes, and one *O*-methyltransferase were also identified. Twenty SM genes were found in the CP-Up module including the PKS gene involved in tenellin biosynthesis, two core genes in a putative lasiodiplodin biosynthetic gene cluster, as well as two *O*-methyltransferases and two NRPS genes.

### Detoxification of host or microbe toxins

The CP-Up module contained a royal jelly protein involved in insect melanization, a hydroxyphenylpyruvate dioxygenase involved in melanin homogentisic acid biosynthesis, as well as three genes involved in antibiotic biosynthesis and three genes involved in antibiotic resistance were identified, along with one serum paraoxonase in quinone detoxification. In the HB-Up data set, an enterotoxin, a beta-lactamase, and a sulfotransferase, the latter involved in detoxification of oxamniquine, were found, along with three nitronate monooxygenases in peroxisome that target toxic nitroalkanes produced by the insect host.

### Antioxidant responses in confronting host defenses

Aspects of glutathione-mediated oxidative stress response pathways were found in both the CP-Up and HB-Up data sets (Tables S3 and S4). Five glutathione *S*-transferases (GSTs), one thioredoxin, and a peroxiredoxin were found in the CP-Up module, whereas one GST (different from that found in the CP-Up module) was identified in the HB-UP data set. In addition, 22 peroxisomal proteins, including the expression of the peroxisomal biogenesis factor PEX11B, were also seen in CP-Up.

### Secreted fungal proteins involved in host nutrition scavenging and suppression of host defense reactions

Entomopathogenic fungi produce a wide range of secretory proteins that target host immune defenses and/or help in the degradation and assimilation of host nutrients. A total of 59 secreted proteins (23.0% of the total) were identified in the CP-Up data set. These included 27 peptidases (18 secreted), 5 secreted cutinases/cellulases, and 5 lipases (four secreted). The HB-Up module contained 64 secreted proteins, including 5 lipases/esterases, 10 peptidases, 7 secreted glycolyl hydrolases/transferases (some potentially involved in cell wall remodeling), as well as 1 secreted lysM effector and a peptidase inhibitor I9 protein that may target host proteases. The HB-Dn module contained 27 secreted proteins, including 9 glycolyl hydrolases/transferases, 3 GPI anchored proteins, 2 peptidases, and 2 small secreted proteins (SSPs). SSPs (≤300 aa) have been shown in several pathogenic fungi (including *B. bassiana*) to serve as key effectors in targeting host processes to increase successful infection (24, 25). A total of 11 and 17 SSPs were identified in the CP-Up and HB-Up modules, respectively. Of those 28 infection-associated SSPs, no clear domain annotation was detected for most using either pfam or InterPro database analyses, except for two members found in the CP-Up data set: a necrosis-inducing secreted protein 1 (Nis1) with homologs in phytopathogenic fungi that induce necrotic lesions in host plants, and one SSP annotated as a CAP-like protein with homology to plant pathogenesis-related protein 1 (GAPR-1), and one SSP found in the HB-Up data set, VLP4, previously characterized as a virulence factor in *B. bassiana* (26). To further investigate these SSPs, a systematic phylogenetic analysis was performed for all the *B. bassiana* SSPs (Table S7). Eleven SSPs (seven in the CP-Up and four in the HB-Up modules) showed the highest similarity to sequences outside of the Sordariomycetes, mainly to Eurotiomycetes and Dothideomyceta. Fifteen SSPs (3 in the CP-Up and 12 in the HB-UP modules) were only found in entomopathogenic fungi, with analogs of 8 SSPs (in HB-Up) only found outside Cordycipitaceae in *Metarhizium* (Clavicipitaceae), with 7 others (3 in CP-Up and 4 in HB-Up) found exclusively in Cordycipitaceae, and further 2 SSPs apparently only found in *B. bassiana* thus far.

### Signal transduction pathways and transcription factors involved in the fungal infection process

In total, 39 transcription factors and 9 kinases were found in four infection-associated subgroups, and 42 of them, accounting for 87.5% in total, were annotated by PHI to be virulence related (Fig. 9C; Table S8). A total of 13 transcription factors (TFs) and 1 protein kinase RIO1 were found in the HB-Up module, including 7 Zn_2_Cys_6_ TFs and 4 MYB TFs. Of these, analogs of two Myb TFs (BDP1, TBF1) that regulate the transcription machinery in *Saccharomyces cerevisiae* were identified, as well as homologs of two C6 transcription factors that regulate growth, conidiation, virulence, and stress responses in *Magnaporthe oryzae* (27), and a homolog of a C6 TF (UME6) in *Candida parapsilosis* linked to fungal virulence (28). The HB-Dn module contained 10 TFs (including 5 Zn_2_Cys_6_, 2 C_2_H_2_, and 2 bZIP TFs) and 3 kinases, including homologs of 2 *S. cerevisiae* TFs (UPC2 and AZF1) that govern sterol uptake and carbon metabolism (29, 30), a homolog of a C6 TF in *Fusarium graminearum* implicated in fungal virulence (31), and a homolog of the bZIP regulator MeaB from *F. oxysporum* that was found to be hypervirulent to tomato after gene deletion (32). The CP-Up module contained 15 TFs (9 Zn_2_Cys_6_) and 3 protein kinases. Of these, homologs to 3 Zn_2_Cys_6_ characterized in *M. oryzae* and found to regulate fungal germination or conidiation (27) were identified. Homologs of two TFs (MET32, UGA3) and OAF3 are also known to regulate amino acid metabolism and oleate metabolism, respectively (33 – 35). In addition, homologs of a homeobox TF and the PDK1 kinase involved in fungal virulence in *M. oryzae* and *F. graminearum,* respectively (36, 37), were identified. A GPCR pheromone receptor (*Ste2*) in the MAPK *FUS3 cascade* was also found in module CP-Up. The CP-Dn data set also contained two putative kinases not previously characterized in fungi.

### Epigenetic and other posttranscriptional modifications during fungal infection

Seven methyltransferases were found in the HB-Up module. Of these, four were predicted to localize to the cell nucleus and two to the cytoplasm. These included one histone methyltransferase, one arginine *N*-methyltransferase, and one lysine *N*-methyltransferase (EFM3) (Table S4). In addition, six GNAT acetyltransferases in charge of protein acetylation were identified in the CP-Up modules and two GNAT acetyltransferases in the HB-Dn data sets. Twenty-five genes (~17% of the total) involved in ncRNA biogenesis were enriched in the HB-Up module, with expression of 17.4% protein members elevated during hemolymph colonization, including PRP3/PRP8 in pre-mRNA splicing, PUS7 for snRNA pseudouridylation, and RPC34/RPC11/DED1/RPC82/BDP1 in RNA polymerase III-mediated transcription of untranslated RNAs. In the CP-Up module, an mRNA decapping hydrolase controlling mRNA maturation was also identified.

## DISCUSSION

### Insights from co-expression analyses leading to increased understanding of the process of fungal infection of insect hosts

In this study, we integrated 76 RNA-seq data sets collected from diverse growth conditions, cell types, and developmental stages, employing a four-step pipeline to generate gene networks to provide specific networks of the gene expression landscape and dynamics in the entomopathogenic fungus *B. bassiana*. In particular, we targeted two stages of the infection process, namely (i) cuticle penetration (4–24 h post-initial contact between the fungus and the insect, CP) and (ii) growth within the insect hemolymph, which occurs post-cuticle penetration and involves a dimorphic switch in the growth of the fungus to free-floating hyphal bodies (HB). Differentially expressed transcripts were further subdivided within these two categories into those showing increased (Up) or decreased (20) expression, with the total number found in each category as follows: CP-Up, 257 transcripts; CP-Dn, 12; HB = Up, 334, HB-Dn, 185. Infection-specific and trait-specific modules were identified using a combination of two clustering algorithms that uncovered the dynamics related to metabolism, signal transduction, virulence, and other factors during pathogenesis as the fungus confronts the host niches of the insect cuticle and hemolymph, respectively.

### Nutrition depletion facilitated host invasion

Entomopathogenic fungi infect insect hosts typically by attaching spores to the surface, followed by the generation of germ tubes and/or appressoria that facilitate the penetration of the host cuticle. The fungus then grows and develops inside the insect body until host death, after which the fungus sporulates on the cadaver (38). Efficient utilization of host nutrients (23) is critical to successful mycosis and the completion of the fungal life cycle (39). The insect cuticle contains a variety of lipids, some of which may be toxic to microbes, as well as the highly insoluble chitin polymer crosslinked to proteins that form a highly sclerotized barrier to many microbes (40). Thus, the fungus has evolved an arsenal of “cuticle-degrading enzymes” that include proteases/peptidases, glycosidases (including chitinases), and lipases/hydrocarbon utilization enzymes (6). Confirming these requirements, our data show that in the cuticle penetration data set, increased expression (CP-Up) of a series of nutrient hydrolysis, uptake, and consequent metabolism-related genes was identified. Significant enrichment of transcripts in terms of membrane proteins and peptidases was noted, including several aspects previously not known. In addition to carbohydrate and amino acid uptake (presumably from the breakdown of host derived chitin and proteins), the CP-Up module included a large set of transporter-mediated uptake of ions, including Ca^2+^, Zn^2+^, and K^+^, suggesting that scavenging for these elements may be critical for successful penetration. In line with carbohydrate and amino acid uptake, pathways involved in amino acid degradation, glycolysis/TCA cycle, and coenzyme biosynthesis were enriched. Similarly, for the *in vivo* generated hyphal bodies that float throughout the insect hemocoel, utilizing nutrients present in the hemolymph (the HB-Up module), enrichment of transcripts for lipases and peptidases. Coupled to distinct (from the CP-Up data set) transporters, for example, uptake systems for amino acids, lipids, and diverse ions, including Ca^2+^, Zn^2+^, Fe, and Mg^2+^, were seen. Metabolically, the HB-Up module was enriched for FAA and carbohydrate metabolism coupled to nucleotide biosynthesis likely facilitating hyphal body proliferation.

### Entomopathogenic fungi employ multiple strategies to counteract with host immune response

Fungal infection results in intense immune responses that include elevated body temperature (behavioral fever), oxidative burst via reactive oxygen species (ROS) production, melanization, hemocyte and fat body activation, phagocytosis, as well as production of antibiotics and mycotoxins (41). In turn, entomopathogenic fungi have evolved means of actively suppressing and/or avoiding/shielding themselves from these defenses (13). For example, *B. bassiana* has been shown to secrete a wide variety of insect toxins, immune-suppressing agents, antioxidants, small RNAs, endotoxins, and ribotoxins that interfere with host immune defense (42, 43). A series of small, secreted proteins (SSPs) are widely known to play key roles in host immune interference, but these have not yet been fully characterized in entomopathogenic fungi. Here, a suite of 11 and 17 SSPs were enriched in the CP-Up and HB-Up data sets, and the elevated expression of some of these was confirmed by qRT-PCR. Phylogenetic analyses indicated that these proteins were uniquely conserved in entomopathogenic fungi or Cordycipitaceae, and the possibility that some of these were acquired via horizontal gene transfer (HGT) events. These findings indicate a new avenue for research on hitherto uncharacterized proteins in entomopathogenic fungi that may play important roles in the evolution of insect pathogenicity. Hyphal bodies are known to undergo significant cell wall remodeling presumably in order to evade host immune systems, which includes altering and/or camouflaging various pathogen-associated molecular patterns (PAMPs), such as cell wall glucans, from host immune recognition and attack (44, 45). Consistent with this, the HB-Up module was enriched in activities related to cell wall modification, including a series of glycoside hydrolases, glycosyltransferases, and cell wall/chitin-binding proteins. A variety of secondary metabolites have been shown to be important for host infection by phytopathogenic and animal fungal pathogens, typically resulting in host cellular toxicity and/or immune suppression (46 – 48). Accordingly, several *B. bassiana* secondary metabolites (49), for example, beauvericin and oosporein, have been shown to be important for various stages of the infection process (50); however, whether these systems are active specifically during hyphal penetration and/or in hyphal bodies has remained obscure. Our data show that both the CP-Up and HB-Up modules contain distinct sets of SM core genes/clusters. In CP-Up, the tenellin cluster as well as putative clusters for echinocandin B1 and lasiodiplodin biosynthesis were identified, whereas in the HB-Up module, the members of the gene clusters for the synthesis of oosporein, a siderophore, and a putative phenalamide A2 were found. However, when compared to the overall transcriptomes from various developmental stages, two interesting points have emerged from our analyses: first, among previously characterized SMs including those for beauvericin, tenellin, and oosporein, these did not appear to be specifically enriched in the CP-Up and HB-Up modules (even when they were differentially expressed between these two data sets). One possible explanation for this is that these SMs are also expressed under noninfection conditions, presumably because they confer some advantage even under saprophytic and/or stress condition growth. Second, a number of the SMs our analyses have identified have not previously been reported to be involved in infection. Further characterization is needed to confirm and/or demonstrate the role(s) of these systems in infection; however, our data have led to new leads and pathways implicated in specific aspects of the fungal infection process.

### Processes of fungal detoxification

During infection, the host microbiome as well as the production of host toxic compounds (including SMs) likely act as significant barriers for the growth and infection of many microbes (51, 52). Detoxification processes can therefore be important aspects of the infection process (53). Production of quinones by some insects, particularly those that show high resistance to fungal infection, for example, the red flour beetle *Tribolium castaneum*, has been shown to be an adaptation that can help reduce susceptibility to entomopathogenic fungi, and while *B. bassiana* possesses enzymes (in this case a hydroquinone reductase) that can (partially) detoxify these quinones, it is not active enough to fully overcome this host defense (7, 54). In addition, *B. bassiana* has been shown to express a variety of cytochrome P450 enzymes, and include production of peroxisomes and associated enzymes in response to host lipids. Consistent with this, the CP-Up modules were enriched in enzymes involved in the degradation of antibiotics and/or insect toxins including quinones, whereas the HB-Up module was enriched in the expression of genes implicated in the degradation of toxic nitroalkanes. As with the SM clusters, several previously characterized cytochrome P450 enzymes and lipases, potentially involved in detoxification were not found in either the CP-Up or the Hb-Up modules, indicating their potential wider biological roles. However, the CP-Up module did contain several (uncharacterized) cytochrome 450 enzymes potentially involved in detoxification of melanization, as well as a suite of antioxidant genes including glutathione *S*-transferases, thioredoxins, and peroxiredoxins. The HB-Up module also contained various ROS detoxification enzymes, although to a lesser extent than what was found in the CP-Up data set, suggesting that ROS responses are less important for the fungal cells at this stage. Mitochondrial processes, that is, energy availability, are known to be important for fungal virulence and survival in potentially host hypoxic conditions (55, 56). Interestingly, the HB-Up module was highly enriched in mitochondrial functioning, containing enzymes involved in the electron transport chain, an array of mitochondrial ribosomal proteins, chaperonins, and mitochondrial localized enzymes implicated in FFA degradation.

### Fungi initiate different genetic responses during different stages of host infection

Our data show that host invasion is a complex and systematic process that involves temporal programs of gene expression specific to various stages of the infection process, providing support for the distinct requirements needed for cuticle penetration, growth within the host, and growth and sporulation on the insect cadaver (40, 57). Analyses of 76 different RNA-seq data sets for *B. bassiana* indicate that the fungus employs diverse regulatory machineries to fine-tune expression of specific gene networks during fungal infection. However, our data also indicate that a wide range of processes important for successful mycosis are also active during other growth conditions, particularly in response to stress and carbon source availability, suggesting overlapping levels of signals and regulatory mechanisms depending upon environmental conditions. This regulation is mediated at the level of signal transduction, activation/repression of transcription factors, and via epigenetic controls (16, 58). Here, we have indexed a set of activated regulators including those involved in epigenetic regulation and signal transduction, and generated regulatory networks concerning cuticle penetration and hyphal body growth/proliferation. This has included the identification of suites of kinases and transcription factors that have not yet been characterized, providing novel candidate for further study and suggesting that much of the regulatory networks involved in fungal infection remain to be fully characterized. In addition, several (again, as yet uncharacterized) GcN5-realted *N*-acetyltransferases (GNATs) and protein methyltransferases that are involved in protein acetylation or methylation posttranscription were identified as highly specific to either the cuticle penetration or the hyphal body growth stages. Finally, our data suggest overlooked aspects of the machinery of RNA biogenesis, especially ncRNA biogenesis, as potentially critical aspects of each of the infection processes examined.

### Conclusions

**Fig 10:**
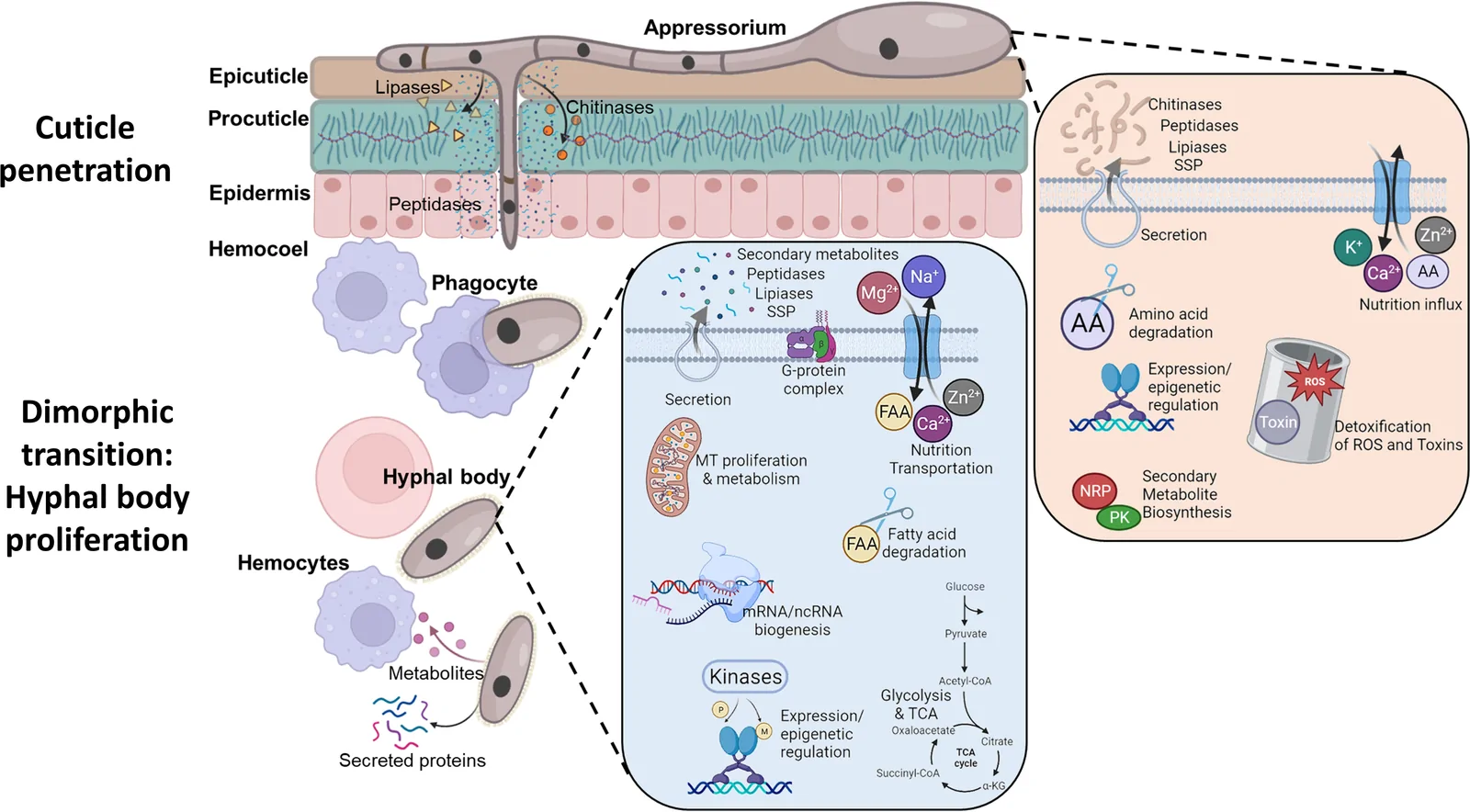
Overview of metabolic and signal transduction processes during infection by entomopathogenic fungi. Insect cuticle comprised of epicuticle, procuticle, and epidermis. Fungal cells attached to the epicuticle germinated to form appressorium that secretes suites of cuticle-degrading enzymes (lipases, chitinases, and peptidases). To overcome barriers of insect cuticle, *B. bassiana* stimulated sets of cellular metabolisms: amino acid degradation, secondary metabolites biosynthesis, detoxification of ROS and cuticle toxins, e.g., quinones, and assumption of nutrition including ions and amino acids, which were orchestrated by the series regulators functions in transcription or epigenetic processes. After penetration into insect hemocoel, fungal cells evade humoral defenses and differentiate *in vivo* into hyphal bodies, which proliferate and secrete metabolites and enzymes (peptidases, lipases, and SSPs) to counter host responses and facilitate nutrition uptake. In hyphal bodies, fungal mRNA/ncRNA biogenesis machinery, members of transcription/epigenetic regulation, and signal sensors of G-protein complex were mobilized, and metabolic processes including fatty acid degradation, glycolysis/TCA cycle, mitochondrial proliferation/metabolism as well as membrane-mediated nutrition transportation (ions, FAA) were activated.

Our co-expression analysis of the entomopathogenic fungus*, B. bassiana*, has integrated a large set of transcriptomic data to provide a focused investigation contrasting cuticle penetration and hyphal body development, resulting in the identification of two discrete infection-associated modules. These data have confirmed previous ideas about these processes as well as provided a large array of previously uncharacterized factors and processes associated with virulence. Aspects of the dynamics of metabolic processes and aspects of the signal transduction networks active in *B. bassiana* at these infection stages were identified, revealing a model in which multiple strategies are employed by the fungus to counteract host defenses and immune responses, threats from competing microbes, and host-mediated adverse stressors (Fig. 10). It is likely that as our understanding of the infection process increases, additional discrete stages and the transcriptional profiles that accompany these stages will be further defined. Our analyses provide first approximation insights into the global genetic networks and mechanisms employed during fungal-insect pathogenesis and identify potential gene targets for further delineating this process and potentially for the development of novel genetic strategies for increasing the efficacy of these insect pest biological control agents.

## MATERIALS AND METHODS

### Strains and culture conditions

Five *B. bassiana* strains [CGMCC 7.34, ARSEF 2860, GXsk series (GXsk1011, CQsk1209, GXtr1009)] were used for generation of the RNA-seq pool. As detailed in Table S1, which includes the cell type, strains, media/growth conditions, and other parameters of the RNA-seq data. For data generated as part of this work, RNA-seq samples were derived from *B. bassiana* strain CGMCC 7.34 (obtained from the China General Microbiological Culture Collection Center). The fungus was routinely grown on various media, including Czapek-dox agar/broth (CZA/B), potato dextrose agar/broth (PDA/B), or Sabouraud dextrose agar or broth (SDA/SDB, Difco Laboratories, Detroit, MI).

### RNA isolation for transcriptomic library construction

Fungal samples for RNA-Seq were prepared as follows: (i) 3-d-old mycelia collected from *B. bassiana* strain CGMCC 7.34 grown in SDB (26°C, with aeration) were processed for RNA extraction for samples LH2/LH20 (WT_SDB/WT_CK) prepared in different sets (59) and hemolymph was collected from *B. bassiana*-infected *G. mellonella* by topical inoculation (48 h post infection), and hyphal bodies were harvested from hemolymph by centrifugation and RNA extracted for sample HB5 (WT_HB) (Table S1). RNA isolation was performed as previously described (60). Briefly, total RNA was isolated using the TRIzol reagent (Invitrogen Life Technology, CA, USA) following the manufacturer’s protocols. Triplicate biological samples were prepared and pooled with unique tags for downstream sequencing. RNA purity and quality were examined by agarose gel electrophoresis and photometric analysis (Pearl nanophotometer, Implen, Munich, Germany). RNA-Seq libraries were generated with the Truseq RNA kit (Illumina, USA) according to the manufacturer’s protocols, then subjected to Illumina HiSeq 2500 (Majorbio, Shanghai, China) RNA sequencing with paired end sequencing mode to generate transcriptomic data with an average sequence length of 150 bp. The raw RNA-seq data were deposited in the NCBI SRA database as the accession series PRJNA865046 and PRJNA962818. Additional RNA-seq raw data sets were collected from NCBI SRA databases with NCBI SRA tools using the program “fastqdump.”

### qRT-PCR

Quantitative real-time PCR (qRT-PCR) validation of the expression of specific genes was performed using cDNA generated using the TRUEscript RT Kit (Aidlab, China). Briefly, conidia of *B. bassiana* CGMCC 7.34 were collected from 5 d SDAY/4 plates and the spore density adjusted to 1 × 10^8^ cells/mL with 100 µL inoculated onto either SDAY plates, 100 mL SDB broth, or 100 mL BS medium containing 2 g peeled-cleared silkworm cuticle homogenized powder (cuticle penetration sample). For *B. bassiana* hyphal body preparation, 2 µL of spore suspension was injected into the hemocoel of second instar larvae of *Galleria mellonella* (30 insect/treatment with three replicates). Fungal cells were collected after 3 d growth on the various media (SDAY/SDB/insect cuticle), and 72 h after injection for the hyphal bodies, and washed with ddH_2_O before RNA extraction. RNA extraction was performed as described previously (60). After RNA quality check by gel electrophoresis, 1 µg of total RNA was reverse-transcribed with primer oligo (dT) following cDNA synthesis kit (TaKaRa Bio, China) to generate the first-strand cDNA for use as the template for PCR amplification. Expression of candidate genes was examined using RT-PCR with primer pairs listed in Table S2. All RT-PCR tests were performed for 25 cycles with expression of the *B. bassiana* β-tubulin gene used as the reference gene according to the manufacturer’s instructions (BioRad) as described previously (60).

### Quality control, genomic mapping, and read extraction of RNA-seq data set

After the collection of *B. bassiana* RNA-seq raw data derived from the various databases and as generated in this study, a transcriptomic data analysis pipeline (61) was generated for determining gene transcript abundances and performing comparative analyses between the different data sets (Fig. 1). Briefly, RNA-Seq data were first subjected to FastQC (v 0.11) (62) for sequencing quality control, then Trimmomatic (v 0.32) (63) was used for sequencing adaptor trimming and removal of unpaired sequences. The Hisat2 (V 2.2.1) (64) program was used for genomic gene transcript mapping to the *B. bassiana* genome (GenBank accession: GCA_000280675.1), after which Samtools (v 0.1.18) (65) and Subread (v 2.0.0) (66) were employed for transcript assembly and gene transcript abundance, in which the transcriptomes were quantified. All analyses were performed on a Tecent cloud server with the Linux system Ubuntu 18.04.4.

### Global gene co-expression analysis

For co-expression analyses, data normalization and clustering were performed using several tools. Transcript counts were first normalized and quantified before co-expression analysis. Briefly, extracted transcript counts from all RNA-seq samples were subjected to the R package edgeR/limma for data transformation to FPKM, RPKM, TPM, upper quantile (UQ) normalized FPKM, and FPKM^t1^ (Log *trans* FPKM + TMM normalization + median polish). The LogCLR was generated in Coseq with log2 transformed, TMM normalized, and median polished. For co-expression clustering, normalized data of LogCLR were subjected to a pair of R-based tools such as Coseq (1.20.0) (67) and WGCNA (21). For Coseq analysis, K-means clustering algorithm was deployed. A range of cluster counts from 2 to 50 was tested for optimal cluster number, and the calculation was done for 50 iterations with parameter settings as: Counts, K = 2:50; transformation = “logclr”; norm = “TMM”; model = “kmeans”). The program was run repeatedly, and the resulting cluster counts with the highest frequency were selected as optimal ones for further analysis. For Pearson correlation (PCC)/hierarchical clustering-based WGCNA analysis, LogCLR data were uploaded and the Pearson correlation between each gene was calculated, after which a weighted adjacency matrix reflecting gene-gene co-expression intensity was generated, hierarchical clustering was conducted, and genes with similar expression profiles were unified as modules, after which a global co-expression network was generated with Cytoscape (v3.9.0) and Gephi (v0.9) with the correlation matrix collected from WGCNA. Two infection process-associated modules were collected from two tools and integrated with a minimum PCC value of 0.6 in between module genes, followed by manual curation according to the coherence of global expression profiles.

### Functional classification of module genes

For general functional classification of module genes, two classic tools, GO and KOG enrichment analyses, were deployed. Briefly, genes were subjected to GO and KOG classification tools using the Omicshare platform (www.omicshare.com) with mapping to annotation databases collected from Uniprot (uniprot.org/) and MycoCosm (mycocosm.jgi.doe.gov/), respectively. In addition, a suite of databases was used for specific analysis, including TCDB (Transporter Classification Database, to March 2023), CZAY (Carbohydrate-Active enZYmes Database, to March 2023), and PHI-base (Pathogen-Host Interactions, v 4.14). The protein sequences in these three databases were all downloaded from given websites (TCDB, https://tcdb.org/; CAZY, http://www.cazy.org/; PHI, http://www.phi-base.org/) and built locally with BLAST+ (v2.13.0). Protein sequences of module genes were then queried against those databases with a *P*-value threshold of 1e-5. The homologous gene annotation was used for functional classification of given categories. Subcellular localization analyses of module genes were performed using deeploc 2.0 (68). For pathway annotation, protein sequences of module genes were mapped to the yeast genome with BLAST+ and subjected to SGD (Saccharomyces GENOME DATABASE) and Metacyc (https://metacyc.org/) for regeneration of metabolic pathways. Protein family annotations of *B. bassiana* in the Uniprot database were used for family protein clustering. The Fungal Secreted Protein (FSD) database (http://fsd.snu.ac.kr/) was used for annotation of secreted proteins in given modules.

### Data availability, analysis, and visualization

For RNA-seq samples in this study, the transcriptome raw data were all stored in the NCBI SRA database with given accession numbers as listed in Table S1. For transcriptomic data similarity analysis, normalized data (LogCLR) were subjected to the R package “ape” (69). For generating a PCA plot with decoration of four sample types, the hierarchical clustering of RNA-seq samples was performed with R package “corrplot”-based PCC values in between samples. Raincloud plot tools in Omicshare platform were employed for illustration of sample PCC relations inside sample groups. For co-expression network visualization, expression matrix (LogCLR) was subjected to function “cor ()” in R package “stats,” PCC matrix of given module genes was first screened with threshold of PCC value of 0.6, then subjected to Gephi (v 0.9) for visualization of co-expression networks with clustering mode of Fruchterman Reingold, gene modules were decorated accordingly. All dot/bubble/heatmap plots in our study were visualized with the R package of ggplot2 and decorated accordingly. Metabolic pathways were illustrated based on Metacyc annotation and manually curated for presentation. For secondary cluster annotation, the *B. bassiana* genome was subjected to antiSMASH online and SM cluster information was mapped to infection module genes, and the global expression of clusters was visualized accordingly. Biorender was deployed for the generation of the graphic model. ORA algorithm was used for enrichment analysis of mapped module genes in three databases (TCDB, CAZY, PHI).

## SUPPLEMENTAL MATERIAL

Supplemental Figures spectrum.01809-23-s0001.docx Figures S1 to S3.

Supplemental Tables spectrum.01809-23-s0002.xlsx Tables S1 to S8.

ASM does not own the copyrights to Supplemental Material that may be linked to, or accessed through, an article. The authors have granted ASM a non-exclusive, world-wide license to publish the Supplemental Material files. Please contact the corresponding author directly for reuse.
