# Supplementary material for: Fungal co-expression network analyses identify pathogen gene modules associated with host insect invasion
Source: Microbiol Spectr. 2023 Sep 1;11(5):e01809-23. doi: 10.1128/spectrum.01809-23 (PMC10581046; doi:10.1128/spectrum.01809-23)
Supplement: Supplemental Figures — Figures S1 to S3. [file spectrum.01809-23-s0001.docx]

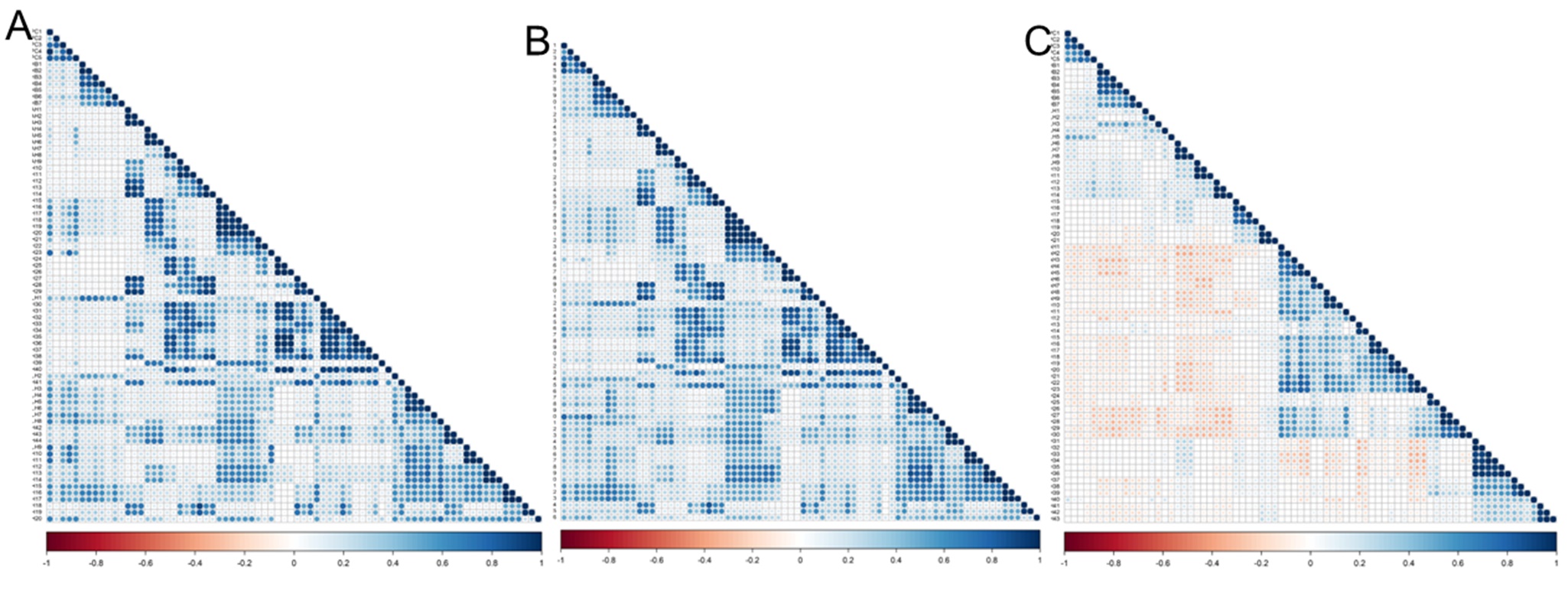


Figure S1 Pearson-correlation (PCC) matrix of collected RNA-samples with different normalization procedures: A: TPM, B: up-quantile Normalized raw counts, C: Transformed FPKM value (FPKM^t1^) as detailed in result section.


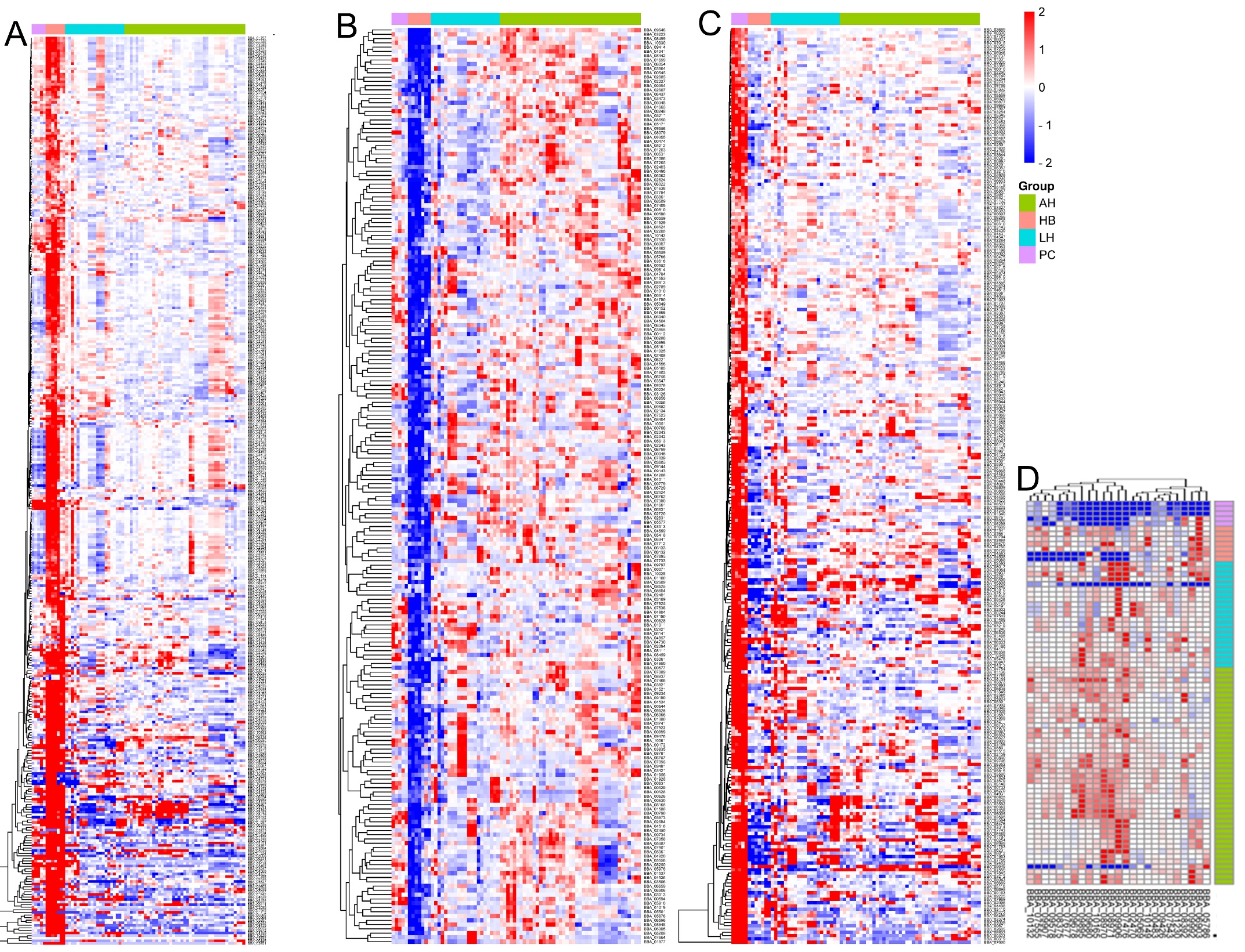


Figure S2 Global expression pattern of 4 infection associated subgroups: A: HB_Up, B: HB_Dn, C: CP_Up, D: CP_Dn. Sample groups were annotated as bar on top with different color. Gene expression of LogCLR were colored red to blue as indicated.


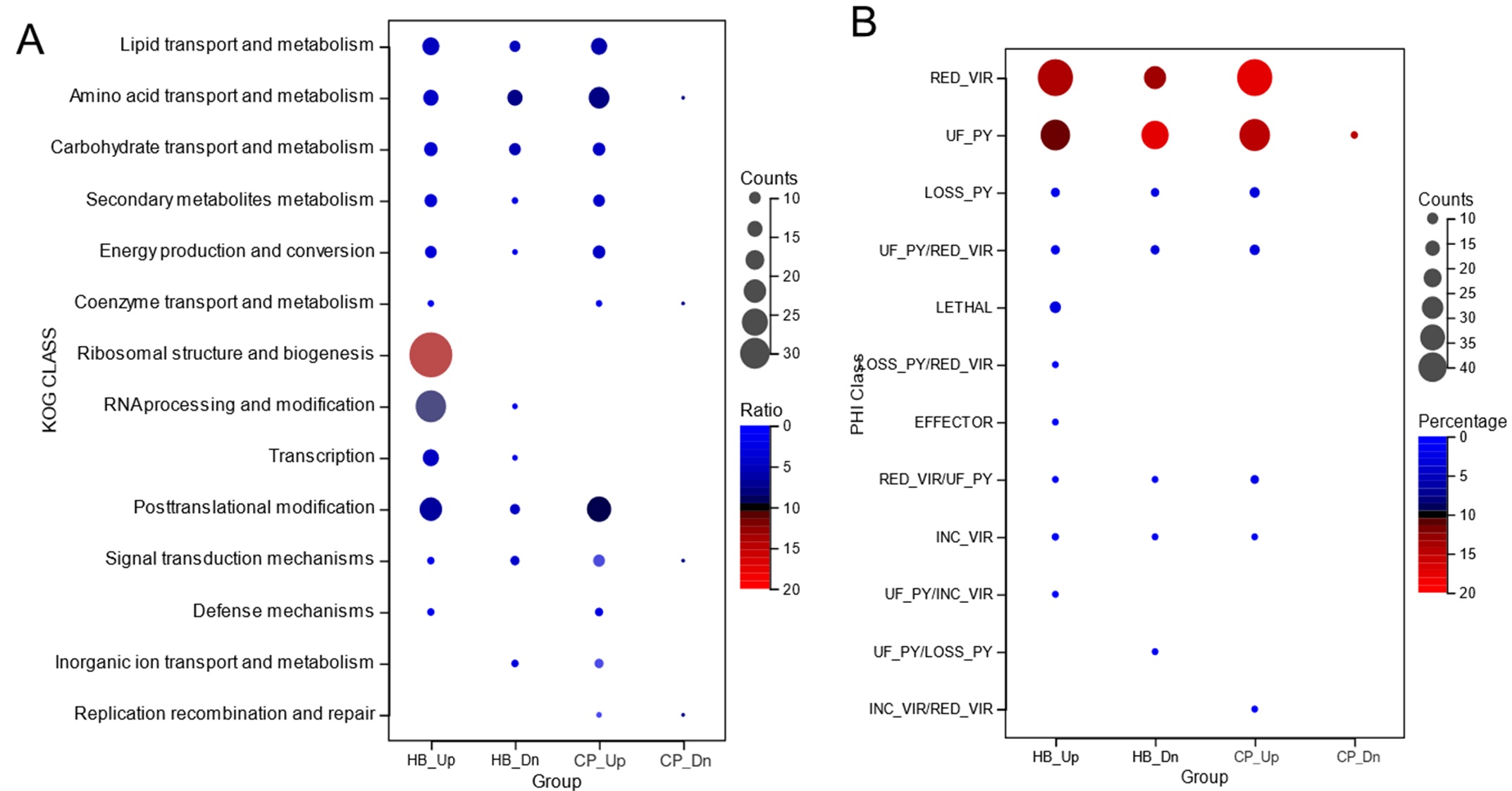


Figure S3 KOG (A) and pathogen-host interaction (PHI) (B) classification of 4 infection associated subgroups. Size of nodes reflects ratio of enriched genes in given modules. Confidence intervals (P-values) were synergy to color bar from blue to red as indicated. Abbreviates: UF_PY: unaffected_pathogenicity, RED_VIR: reduced_virulence, LOSS_PY: loss_of_pathogenicity, INC_VIR: increased_virulence_(hypervirulence).
